# Population Genomics and the Environmental Drivers of Population Structure in a Cosmopolitan Marine Predator, *Tursiops truncatus*

**DOI:** 10.1111/mec.70182

**Published:** 2025-12-01

**Authors:** Daniel M. Moore, Andre E. Moura, Ada Natoli, Elena Papale, Emily G. Cunningham, Mónica A. Silva, Tilen Genov, Stefania Gaspari, Giuseppa Buscaino, Per Berggren, Darren R. Gröcke, A. Rus Hoelzel

**Affiliations:** ^1^ Department of Biosciences Durham University Durham UK; ^2^ Centre for Ecology and Conservation, Faculty of Environment, Sustainability and Economy, University of Exeter Cornwall UK; ^3^ Department of Genetics and Biosystematics Faculty of Biology, University of Gdansk Gdansk Poland; ^4^ Zayed University, College of Natural and Health Science Abu Dhabi UAE; ^5^ UAE Dolphin Project Dubai UAE; ^6^ Bioacoustics Lab, Institute for the Study of Anthropic Impacts and Sustainability in the Marine Environment, National Research Council (CNR‐IAS), Capo Granitola Trapani Italy; ^7^ OKEANOS—Institute of Marine Sciences, University of the Azores & IMAR—Institute of Marine Research Horta Portugal; ^8^ Morigenos—Slovenian Marine Mammal Society Piran Slovenia; ^9^ Department of Biodiversity, Faculty of Mathematics, Natural Sciences and Information Technologies, University of Primorska Koper Slovenia; ^10^ Consiglio Nazionale Delle Ricerche‐Istituto di Scienze Marine Ancona Italy; ^11^ School of Natural and Environmental Sciences, Newcastle University Newcastle upon Tyne UK; ^12^ Department of Earth Sciences Durham University Durham UK

**Keywords:** genomics, phylogeography, population structure, stable isotopes, *Tursiops truncatus*

## Abstract

The marine environment comprises vast regions without physical barriers to movement, making the understanding of population isolation and the evolution of diversity challenging. This is especially the case for highly mobile marine species. Here we investigate populations of the common bottlenose dolphin ( *Tursiops truncatus* ) across the Mediterranean Sea and adjacent North Atlantic using high‐resolution genomic markers (RADseq) and stable isotope analyses to better understand the evolution of population structure in this system. High‐resolution genomic data and broad geographic sampling revealed patterns of structure not previously identified, and integration with stable isotope data suggests that prey choice varies across this region. Unexpected patterns included genetic and isotopic similarity between the North Atlantic and the region around Sicily (but not including the medially located Gulf of Cádiz and surrounding regions). The regional habitat within and beyond the Mediterranean Sea is structured with ocean frontal systems including thermal and halocline transitions, several of which show alignment with genetic transitions within our data. Our data help to distinguish among possible drivers of population differentiation for a marine predator that has the potential for long‐distance dispersion.

## 1 Introduction

Understanding the processes that lead to the differentiation of lineages is fundamental to understanding evolution. It is clear that genetic drift and natural selection can differentiate populations in isolation. However, it is less well established how differentiation is promoted when physical boundaries (such as mountain ranges or rivers in terrestrial environments) are less well established or absent altogether, as in the marine environment. This is especially the case for highly mobile species that have high dispersal potential (see Hoelzel 1994). Here we investigate regional populations within the Mediterranean Sea for a species that has the potential for long‐distance dispersal. There are, however, environmental distinctions between separate regions within the Mediterranean, in particular between the eastern and western basins. We integrate population genomic and environmental data to test hypotheses about possible ecological drivers of population differentiation in this system.

The common bottlenose dolphin *Tursiops truncatus* (Montagu, 1821) is a highly social species with an almost global distribution, being absent only from polar waters (Wells et al. 2019). It shows population structure throughout its range, especially among nearshore populations (see IWC 2018). In the North Atlantic, genetically differentiated coastal populations have been reported from the UK to Gibraltar (Parsons et al. 2002; Natoli et al. 2005; Martinho et al. 2014; Nykänen et al. 2019). Population structure within the Mediterranean region has been reported for *T. truncatus* , suggesting divergence break points at the Bosphorus, the Siculo‐Tunisian Strait, and the Almeria–Oran front in the Alboran Sea (Natoli et al. 2005) and within the Adriatic (Gaspari, Holcer, et al. 2015), based on microsatellite DNA and mtDNA. It has been suggested that coastal populations within the Adriatic have experienced a recent genetic bottleneck with evidence of gene flow from the Ionian Sea and possibly other Mediterranean regions (Galov et al. 2011). Post‐glacial expansion of offshore populations into coastal areas during periods of sea level rise has been suggested as a probable cause of this gene flow, as well as a mechanism for the founding of coastal populations (Gaspari, Scheinin, et al. 2015; Moura et al. 2020).

Population structure within the Mediterranean basin based on non‐genetic metrics has also been reported. For example, it has been suggested that the reported dwarfism in the Levantine Basin off Israel (‘Levantine nanism’, also reported for fish and invertebrates) in *T. truncatus* may be a product of genetic isolation (Sharir et al. 2011). Concurrence of acoustic with genetic data was found in the Macaronesian *T. truncatus* (Papale et al. 2014), and it has now been demonstrated that *T. truncatus* found in the Strait of Sicily in the Mediterranean Sea also shares characteristics of whistle communications with the Macaronesian population (La Manna et al. 2017).

It is well established that *T. truncatus* exhibits differential niche specialisation in the form of at least two ecotypes: offshore and coastal (Hoelzel et al. 1998; Lowther‐Thieleking et al. 2015; Perrin et al. 2011; Rossbach and Herzing 1999). Stable Isotope Analysis (SIA) has demonstrated exploitation of different food resources between offshore and inshore ecotypes throughout the species range (Barros et al. 2010; Dıaz‐Gamboa 2003; Segura et al. 2006) and studies of their teeth show morphological divergence that is consistent with the differential prey targets as suggested by SIA (Perrin et al. 2011). It has been shown that offshore and inshore populations can also be distinguished genetically (Hoelzel et al. 1998; Torres et al. 2003; Fruet et al. 2017). This level of differentiation, both phenotypic and genotypic, has led some to suggest that ecotypes could form valid parapatric subspecies or even species in some regions (Wickert et al. 2016; Costa et al. 2016, 2022).

Aside from the dietary differences between ecotypes, there is evidence of variation in diet specialisation between populations, even sympatric ones (Fernández et al. 2011). In general, the diet of *T. truncatus* is dominated by fish and cephalopods, an observation made in a variety of populations worldwide (Barros and Wells 1998; Blanco et al. 2001; Gladilina and Gol'din 2014; Pate and McFee 2012; Santos et al. 2001, 2007). Considerable local variation in diet has been demonstrated throughout the Mediterranean and Black Sea region by stomach contents analysis and/or SIA (Santos et al. 2001, 2007; Bearzi et al. 2009; Gladilina and Gol'din 2014; Scheinin et al. 2014; Pedà et al. 2015; Bräger et al. 2016; Giménez et al. 2017; Borrell et al. 2006, 2021).

It is likely that niche specialisations and prey choice may be drivers of ecotype differentiation in bottlenose dolphins (Louis et al. 2014; Segura‐García et al. 2018), and thus a major evolutionary driver more broadly. Concurrent genetic and prey choice differentiation has also been seen in other marine predators (e.g., Wolf et al. 2008; Jeglinski et al. 2015; Westbury et al. 2023). However, few studies have sought to analyse data on genetic differentiation and prey choice in an integrated manner.

In this study, we integrate high‐resolution population genomic analyses (based on ddRADseq—see Peterson et al. 2012) with environmental metrics, including stable isotopic signatures for genotyped individuals, to investigate the potential environmental drivers for population structure within the Mediterranean Sea. We reveal previously unreported patterns of population structure within the basin, and correlate this with stable isotope signatures, suggesting that genetically differentiated populations over a small geographic range within this region also specialise with respect to prey resource. We consider these findings in the broader context of the evolution of diversity among populations of a highly mobile apex predator.

## 2 Materials and Methods

### 2.1 Sample Collection and Extraction

Samples included archived DNA (*n* = 131) that had been used in previous studies (Gaspari, Scheinin, et al. 2015; Gaspari, Holcer, et al. 2015; Moura et al. 2013; Natoli et al. 2004, 2005), new biopsy samples (*n* = 8) collected in Sicily, Italy, archive samples from the Marine and Environmental Sciences Centre, Azores (*n* = 30; all of which were collected by biopsy sampling) and stranded animal samples from the University of Valencia (*n* = 8). New biopsy sampling for this study was conducted using a Petron Stealth Wood Stock Crossbow with a 150 lb. draw‐weight and custom biopsy bolts and tips obtained from Ceta‐Dart V/Finn Larsen. All biopsy tips were sterilised with 100% ethanol and flamed prior to use, and groups containing mother/calf pairs were avoided. The sampling protocol followed the Northeast Fisheries Science Centre Cetacean Biopsy Training Manual (Wenzel et al. 2010) and protocols described in Genov, Jepson, et al. (2019). Biopsy sampling in Sicily was conducted under a permit granted by *Ministero dell'Ambiente e della Tutela del Territorio e del Mare* (No 33969) and samples were transported to Durham University under authorisation from DEFRA (IMP/GEN/2014/06). Final sample geographic distributions (archived and new biopsy) are shown in Figure S1 (specific locations of samples) and Figure S2 (numbers per region).

A standard phenol‐chloroform extraction protocol was used to isolate DNA, following Hoelzel et al. (1998). Concentration of DNA extractions was measured using a Qubit 2.0 fluorometer (Invitrogen). DNA extraction quality was assessed using a Nanodrop spectrophotometer (Thermo Scientific) based on OD 260/280 ratios.

### 2.2 Library Preparation and Sequencing

Genomic libraries for double‐digest restriction associated DNA (ddRAD) were constructed following Peterson et al. (2012). For each sample, 500 ng of DNA was digested with MspI and HindIII (New England Biolabs) at 37°C overnight in a 50 μL reaction and then ligated with P1 and P2 adapters. Pools were cleaned using calibrated streptavidin coated SpeedBeads (Sera‐Mag). The concentration of cleaned DNA pools was determined using a Qubit 2.0 fluorometer (Invitrogen). DNA fragments were size selected to a desired length of 325–475 bp on a Pippin Prep (Sage Science). Size‐selected pools were then amplified, and unique pool indices added via PCR. Pool concentration was quantified using qPCR and a commercial quantification kit (Kapa Biosystems). Final libraries were sequenced on three lanes of an Illumina HiSeq 2500 (125 bp paired end reads) at the DBS Genomics facility at Durham University.

### 2.3 Stable Isotope Analysis

All tissue samples were kept frozen at −20°C prior to preparation for SIA. Available tissue samples were derived from multiple sources (pre‐existing archive and fresh biopsies) and tissue types, including skin, muscle and kidney. Due to a concern for bias in δ^13^C values originating from tissues containing large quantities of naturally occurring lipids, all samples were lipid extracted prior to analysis. Samples were defrosted and approximately 0.5cm^3^ was transferred to a clean pre‐labelled 1.5 mL Eppendorf tube. Then, 1000 μL of 3:1 dichloromethane: methanol was added to the Eppendorf before sonication for 15 min. Following this the Eppendorf was centrifuged at 3000 rpm for 10 min before the dichloromethane: methanol mix was carefully removed. These steps were then repeated twice more. Upon removal of the final mix the sample was placed in a drying oven heated to 45°C until the sample was fully desiccated.

Desiccated samples were ground to a fine powder and 0.3–0.5 mg loaded into tin capsules for stable isotope analysis. Analysis for carbon and nitrogen isotopes was performed at the Stable Isotope and Biogeochemistry Laboratory (SIBL), Durham University, using an ECS 4010 Elemental Analyser (Costech) connected to a Delta V Advantage Isotope Ratio Mass Spectrometer (Thermo Scientific). Correction of carbon isotope ratios for ^17^O contribution is reported in standard delta (δ) notation in per mil (‰) relative to Vienna Pee Dee Belemnite (VPDB). Nitrogen isotope ratios are reported relative to atmospheric nitrogen (AIR). Isotopic accuracy was monitored through routine analyses of in‐house standards, which were stringently calibrated against international standards (e.g., USGS40, USGS24, IAEA‐600, IAEA‐CH‐3, IAEA‐N‐1, IAEA‐N‐2), giving a total linear range in δ^13^C between −46‰ and +3‰, and between −4.5‰ and +20.4‰ for δ^15^N. δ^13^C and δ^15^N analytical uncertainty was typically ±0.1‰ (1 SD) or lower for replicate analyses of the international standards and < 0.2‰ (1 SD) for replicate sample analysis. Seventy‐six samples had tissue suitable for stable isotope analysis.

### 2.4 Sequence Analyses

Raw sequencing reads were demultiplexed and quality filtered using the process radtags subprogram in Stacks v1.35 (Catchen et al. 2013). Process radtags flags were defined as *‐q 10 ‐t 92 ‐r –renz_1 msp1 –renz_2 hindIII ‐E phred33*. The *Tursiops truncatus* reference genome TurTru1.96 was downloaded from the Ensemble database (GenBank Accession GCA_003314715.1) was downloaded from NCBI and reference indexes created using the bowtie2‐build command within Bowtie2 v2.2.5 (Langmead and Salzberg 2012). Bowtie2 v2.2.5 was then used to align all sequence reads to the reference genome using default settings (including end‐to‐end alignment). Process_radtags was set to remove reads with low quality scores. Single nucleotide polymorphism (SNP) detection was then completed using the Stacks v1.35 (Catchen et al. 2013) *ref_map.pl* pipeline by manually working through each of the pipeline modules (gstacks and populations), allowing for greater control of quality filters. gstacks was set to remove PCR duplicates (‐‐rm‐pcr‐duplicates) and the SNP discovery alpha threshold was set at 0.05. Minimum PHRED‐scaled mapping quality for considering a read was set to 10 and minimum depth of coverage was set at 3. Further filtering was done within the module Populations. The minimum number of populations where a locus must be present in order to be processed further was set at 6. The minimum percentage of individuals in a population required to process a locus for that population was set at 80%. The module Populations was set to output one SNP per locus (*‐‐*write‐single‐snp). The population map for ref_map.pl. was user‐defined and populations were assigned according to geographic sampling location (Figure S2), based on inference from Natoli et al. (2005) to define population boundaries. Any samples with less than 900,000 reads retained after demultiplexing, were excluded from the population map and thus not used in any further analysis (due to the limiting effect they would have on SNP number). The GenePop file generated using the above pipeline in the Populations module of Stacks v1.35, was then examined for samples with more than 30% missing data (*n* = 6), which were then removed from further analysis. Maximum depth was filtered to 2 times the average depth using vcftools (Danecek et al. 2011), while minimum allele frequency was filtered to > 0.01 using dartR (Gruber et al. 2018).

Initial identification of loci putatively under selection was carried out using the software package Lositan (Antao et al. 2008). Lositan was run with the following parameters: 50,000 simulations, a confidence interval rate of 0.95, a false discovery rate of 0.05, an Infinite Alleles mutation model and a subsample size of 30. The Lositan output table was used to identify loci falling outside of outlier neutrality thresholds, which were selected to form an outlier whitelist for input into the module Populations. Loci that fell below the outlier thresholds were designated as either neutral or balancing and both were included to form a neutral/balancing whitelist for input into the module Populations. Lositan outlier selection was visualised in *R* (R Core Team 2019) using the package ggplot2 (Wickham et al. 2016). Outlier analysis was repeated using OutFLANK (Whitlock and Lotterhos 2015). For OutFLANK, we estimated the null neutral Fst distribution by discarding loci with heterozygosity lower than 0.01. The *F*_ST_ trim proportions for the distribution tails were set to LeftTrimFraction = 0.3 and RightTrimFraction = 0.05. Those values were identified as generating an *F*_ST_ distribution closest to a Chi‐square distribution. The significance threshold was set to *q* = 0.05. Population structure analyses were implemented on both those loci identified as under positive selection and those determined as neutral/balancing to allow for inference on the effects of environmental adaptation on genetic structure. Only outliers that were supported by both Lositan and OutFLANK were retained for outlier analyses.

Population structure analyses were conducted by Principal Component Analysis (PCA; Jolliffe 2011) and Discriminant Analysis of Principal Components (DAPC) implemented in adegenet (Jombart et al. 2010), Admixture (Alexander et al. 2009) with *K* = 6 (based on results of SNMF), SNMF (sparse nonnegative matrix factorisation) implemented using the Landscape and Ecological Studies (LEA) package (Frichot and Francois 2015) with 5 repetitions, 10,000 iterations and a burn‐in of 5000 for 2–8 K. Further structure analysis was undertaken using a Bayesian assignment test for individuals belonging to predefined clusters based on Minor Allele Frequency (MAF) using the *R* package SambaR (de Jong et al. 2021). All of the aforementioned analyses were implemented in *R* (R Core Team 2019). Analysis of contemporary migration rates (gene flow) was calculated using *BayesAss3‐SNPs* (Mussmann et al. 2019) with 1,000,000 iterations including a burn‐in of 100,000 and visualised in *R* using the package Circlize (Gu et al. 2014), following the workflow presented in (Sander et al. 2014).

Identification of the strongest restrictions in gene flow between populations was calculated using Barrier v2.2 (Manni et al. 2004), using matrices of Nei's genetic distances and estimated central latitude and longitude of each putative population based on sample collection sites. Barrier projects a Delauney triangulation network between nodes set on a localised geographical sample area matrix and then estimates node boundaries with the greatest barriers to gene flow based on Monmonier's maximum‐difference algorithm. Analysis of genetic diversity (genome‐wide heterozygosity, Minor Allele Frequencies (MAF) calculations etc.) was conducted using SambaR. Analyses of genetic differentiation (F_ST_) between populations were conducted using the R package hierfstat (Goudet 2005), with significance based on 10,000 permutations. Various sub‐divisions were tested as putative populations by this method (see below). DAPC clusters were found using the function find.clusters with the maximum clusters (max.n.clust) set above the a priori hypothesised number of populations under study (*n* = 6). 100% of the total variance expressed comes from the retained axes of the PCA analysis.

Isoscape plots were produced using Ocean Data View (Schlitzer 2018). Stable isotope (SI) values were projected with DIVA (Data‐Interpolating Variational Analysis—Troupin et al. 2012) gridding and an x‐scale and y‐scale length of 60 and 123, respectively. Investigations of the potential influence of environment and diet on population structure were conducted using redundancy (RDA) analysis implemented with the *R* package Vegan (Oksanen et al. 2010). Data on Sea Surface Temperature (SST) came from CNR‐Med satellite data (Nardelli et al. 2013). Salinity data were derived from the Copernicus Marine Environment Monitoring Service (von Schuckmann et al. 2016). Chlorophyll A data were CNR processed SeaWiFS satellite data (Gregg and Casey 2004). Environmental data were downloaded for 2010–2015 (1997–2002 for Chlorophyll A data), where available and summarised as yearly means. Final data points for each individual were determined based on the geographical location of its genetic sample retrieval. For the RDA analysis samples that had genetic data but no individual SI values were assigned their regional average value for SI.

To estimate historic demography for local populations the Site Frequency Spectrum (SFS) was calculated using ANGSD (Korneliussen et al. 2014). Input data were individual Bam files, and the corresponding reference genome was in fasta format. The site allele frequency likelihood was calculated based on individual genotype likelihoods assuming HWE. Flags were set as follows: GL 1 ‐fold 1 ‐minMapQ 20 ‐minQ 20 ‐minInd 20. SFS was folded and generated as one per population. Sequence length used in SFS derivation was estimated from the Stacks ‘Populations’ file. This was then used in SambaR (de Jong et al. 2021) which utilises the Java‐implemented Stairway_plot_v2 function (Liu and Fu 2020) to derive the visual output based on a bash script blueprint input file. Mutation rate and generation time were both derived from available literature with mutation rate set at 1.5 × 10^−8^ substitutions/nucleotide/generation (Moura et al. 2014) and generation time at 21.5 years (Taylor et al. 2007).

## 3 Results

A total of 176 *T. truncatus* individuals were sequenced for this study. Ten samples were removed from further study due to a low number of reads (< 900,000) and nine further samples were removed due to high levels of missing data (> 30% missing data) (see Figure S3). After filtering there were 2641 neutral SNPs with 1 SNP per paired‐end read from 157 individuals. Average read depth was 295. Outlier detection, implemented in Lositan and OutFLANK (Figure S4), discovered 72 loci putatively under positive selection, identified by both methods. All other loci were considered neutral for the purposes of our analyses.

### 3.1 Genetic Diversity

Genomic estimates of population diversity calculated in R are shown in Figure S5. Briefly, EastMed was most diverse by these analyses, and the Black Sea was least diverse, with all other populations showing similar diversity to each other. The range of heterozygosity across loci was in the expected range for each population with no high outliers (Figure S5B). All populations showed an excess of rare alleles (Figure S5D), suggestive of a post‐bottleneck expansion or selective sweep.

### 3.2 Investigating Population Structure

PCA for neutral markers (Figure S6A) revealed overlapping distributions for CentralMed, EastMed and WestMed. The Azores formed a tight cluster, overlapping with CentralMed samples originating from Sicily. Running PCA with outlier loci revealed a similar spatial patterning. Discriminant Analysis of Principal Components (DAPC), performed on neutral loci and retaining 80 principal components, produced a similar pattern (Figure S6B). Figure S6B reveals a distinct cluster made up of Azorean samples, overlapping with another cluster of Sicilian and Valencian samples.

Pairwise *F*_ST_ values (Table 1) were highest between the Black Sea and the Azores while the lowest value was observed between CentralMed and the WestMed, which includes all of eastern Spain. Despite ordination overlap, *F*_ST_ values among Sicily, Valencia (Spain) and the Azores were all significantly greater than zero. Neutral markers showed less genetic differentiation between a priori populations (Table 1, lower diagonal) than outlier loci (Table 1, upper diagonal). However, population differentiation patterns were consistent across both sets of loci, with Black Sea *T. truncatus* remaining the most distinct population group.

**TABLE 1:** Geographic pairwise *F*_ST_ values calculated using *hierfstat* and based on neutral *loci* (below the diagonal) and outlier *loci* (above the diagonal).

	Azores	Cadiz	West Med	Central Med	East Med	Black Sea
Azores	NA	0.1036	0.1462	0.1681	0.2394	0.5884
Cadiz	0.0238	NA	0.0532	0.0989	0.1817	0.5495
West Med	0.0186	0.0062	NA	0.0319	0.102	0.5543
Central Med	0.0193	0.0121	0.0083	NA	0.0707	0.5575
East Med	0.0287	0.0195	0.0167	0.0066	NA	0.512
Black Sea	0.0482	0.0278	0.0261	0.0277	0.0246	NA

*Note:* All values are significant at 0.05 after Bonferroni correction.

**FIGURE 1:**
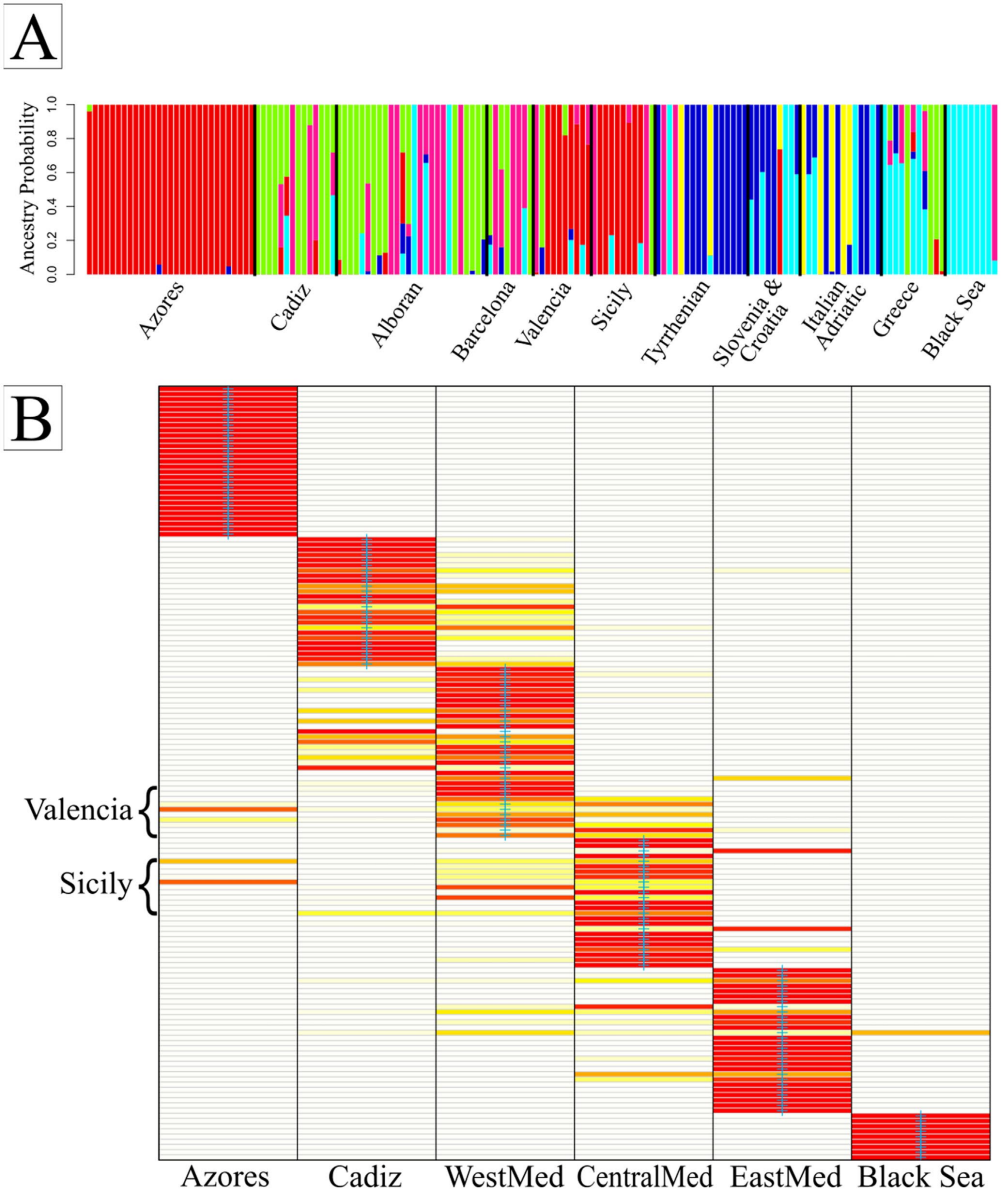
(A) Estimated proportions of each individual's genome (admixture coefficient) that derives from hypothetical ancestral population ‘*K*’ (for *K* = 6). Estimates developed in *Admixture* and visualised in *R*. (B) Assignment probability plot of individuals from DAPC analysis. The *X*‐axis represents the possible assignment populations whereas the *Y*‐axis represents the individual samples. Probabilities are represented by colour with red being high assignment probability, white being low and yellow being intermediate. The Valencia and Sicily samples are highlighted on the *Y*‐axis and this figure clearly shows likely assignment to the Azores population for some individuals.

Investigations of population structure using SNMF in LEA were assessed for *K* from 2 to 8 (Figure S7). Although *K* = 6 was most supported, based on no further structure being revealed with further values of K, there is considerable cross assignment among regions. The proportions of the coefficient of admixture of each *T. truncatus* individual's genome that originated from population clusters for *K* = 6, 6 were more clearly estimated in Admixture (Figure 1A). The Black Sea clustered as a single population, consistent with the *F*_ST_ analysis (Table 1). Individuals from the Ionian Sea in Greece were also differentiated, with some admixture from the Adriatic, Tyrrhenian and Black Sea. Dolphins from Croatia and Slovenia clustered together in a distinct cluster from Italian Adriatic individuals. The DAPC analysis separated the six putative populations, with some admixture among them (Figure 1B). Within the Tyrrhenian Sea (CentralMed) there were some individuals that appeared to be migrants, with strong admixture signals indicating origin from the East Mediterranean (seen for both Admixture and DAPC analyses). Admixture again showed apparent shared ancestry between individuals from the Azores, Valencia and Sicily. This pattern was strongly defined for all levels of *K* investigated from 2 to 8 and is consistent with our PCA, DAPC and *F*_ST_ analyses. This is further demonstrated by DAPC assignments (Figure 1B), with many samples from the Sicily and Valencia regions showing a high probability of assignment to the Atlantic population. Black Sea individuals cluster with the EastMed population. The probability that an individual belongs to a given a priori population based on the Bayesian assignment method in SambaR showed support for pre‐assigned regional populations (Figure S8), but this analysis includes sample origin as a prior.

**FIGURE 2:**
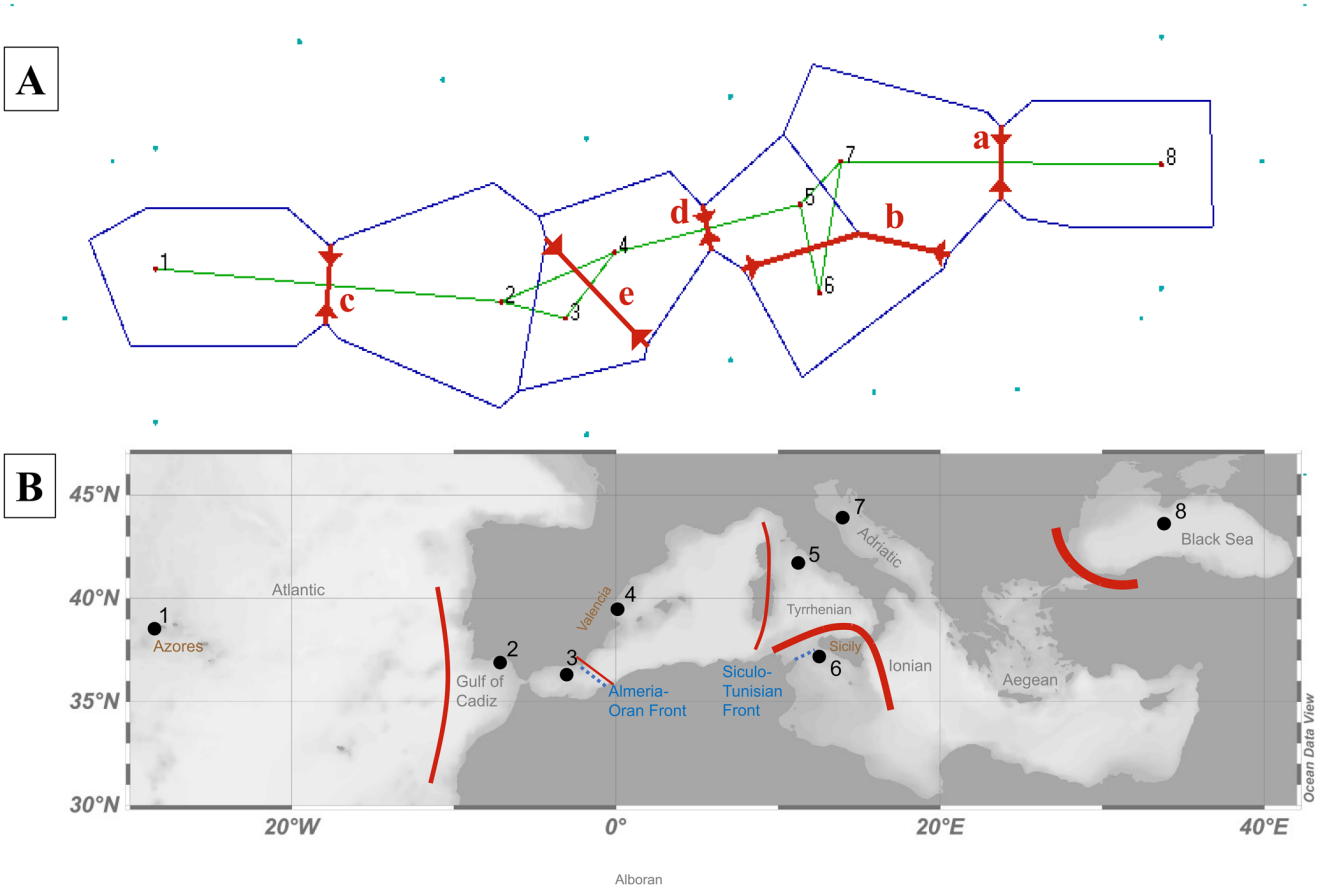
(A) Projection of Delauney triangulation network between localised population nodes (red dots) with the 5 strongest restrictions in gene flow (labelled in order, a–e), as identified through Barrier v2.2, highlighted by the red lines (Top). Green lines represent potential gene flow avenues to nearest neighbour populations. (B) Shows these results projected on to a geographical map with barriers highlighted in (A) fitted to likely real‐world positions. Location numbers indicated in A are shown on the map in B. Key sample locations, sea names and important front regions are labelled.

Population structure was also assessed using Barrier v2.2 (Figure 2A,B). Barrier revealed that the strongest restriction in gene flow was between the Black Sea and all other populations, consistent with our results from Admixture and DAPC (Figure 1). Barrier analysis also highlighted the separation of Sicilian samples from the rest of the Mediterranean as well as an East–West division, possibly focused along the Corsica‐Sardinia alignment. There was a restriction in gene flow between the Azorean samples from the Atlantic and those found in the coastal waters of the Gulf of Cadiz. A fifth restriction to gene flow was also discovered that co‐aligned with the Almería‐Oran front.

Investigations of contemporary migration were conducted with BayesAss3‐SNPs and visualised as circos plots (Figure S9A) and the migration estimates are given in Table S1. Initial examination using a priori population assignments revealed high levels of gene flow from WestMed to all other populations. Secondary levels of gene flow were high from CentralMed and Azores to other populations, though this was still relatively small when compared to the primary geneflow from WestMed. To assess if some of the patterns in Figure S9A were influenced by further population substructure, the analysis was re‐run separated into 9 regional populations (Figure S9B). These were chosen in part based on the assignment analyses, but also geography and to maintain a sufficient sample size. Though gene flow was apparent in all directions, the greatest flow was outward from Sicily. Gene flow estimates are given in Table S2.

Historical demographies of 9 putative populations are presented in Figure S10a–i. However, further structure revealed in other analyses may affect the profiles generated from allele frequency spectra data for these groupings, perhaps especially in the Adriatic sample set. All populations within the Mediterranean apart from Greece show population decline since the last interglacial (Eemian). The Black Sea appears relatively stable, while the Atlantic suggests a population increase over that time. Note that the more recent trends (more recent than ~10,000 years), are likely to be less accurately represented by this method (see Heller et al. 2013).

### 3.3 Exploration of Environmental and Dietary Factors Influencing Population Structure

Stable isotope values were available for 76 individuals (not including individual estimates based on regional values) and came from multiple tissue types (see Table S3). To correct for this, samples from the same location (Azores—Figure S11) with two tissue types, skin (*n* = 6) and muscle (*n* = 6), were tested for differentiation to determine the need to calibrate data based on tissue type. Values for δ^15^N did not meet parametric test assumptions (Anderson‐Darling test, *p* = 0.041) so they were tested with the non‐parametric Kruskal‐Wallis test. Medians were not significantly different (Kruskal‐Wallis, *p* = 0.631). Values for δ^13^C met parametric assumptions (Anderson‐Darling test, *p* = 0.715 and *p* = 0.154 for skin and muscle respectively, confirmed with Levene's test, *p* = 0.824) and were also found to be not significantly different (*t*‐test, *p* = 0.297). Sample sizes for this test were small and multiple or unknown tissues were included from other regions, which complicates inference (Table S5). However, isoscape maps produced using SIA data from all samples (Figure 4) gave a similar pattern of data projection as those isoscape maps that were made conservatively, using SIA data derived only from skin samples (*n* = 29; Figure S12). Therefore, we consider that the inclusion of different tissue types did not bias the biological interpretation of our SIA results.

**FIGURE 3:**
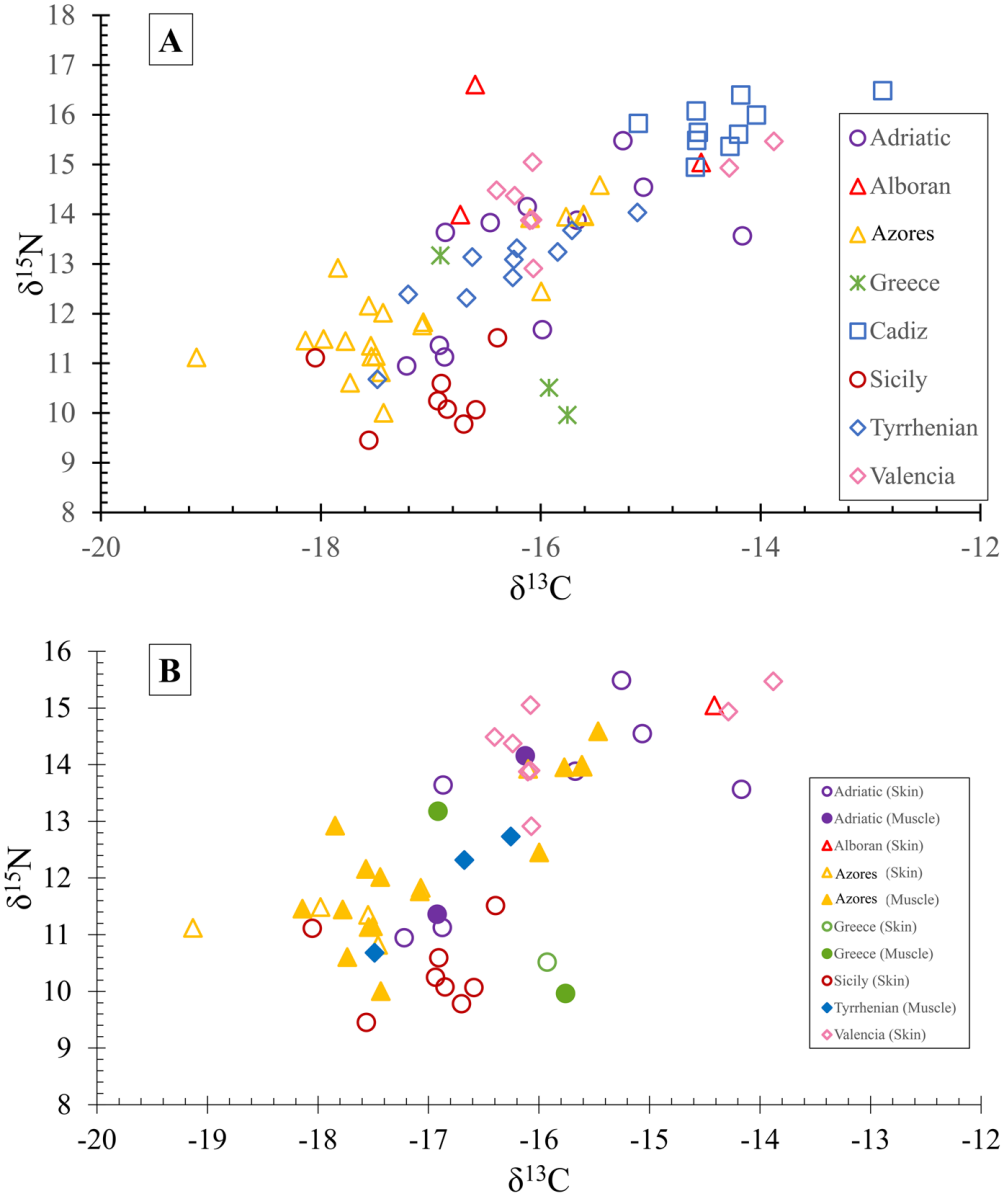
(A) δ^13^C values versus δ^15^N values for all samples, including a mix of tissue types. Values closer to the top right would indicate feeding at a higher trophic level than those found at the bottom left. (B) δ^13^C values versus δ^15^N values for skin and muscle samples only. Skin samples are represented by open symbols whereas muscle samples are represented by closed symbols.

When δ^15^N values are plotted against values for δ^13^C (Figure 3) there is an indication that *T. truncatus* individuals from the Gulf of Cádiz were feeding at a higher trophic level than other groups. By contrast, most Azorean and Sicilian *T. truncatus* were feeding at a lower trophic level. Most other populations had significant intra‐population variation with individuals positioned over a broad spectrum of trophic levels. *T. truncatus* from Greece appears to be feeding at a similar trophic level to Azorean individuals but may have less negative δ^13^C values due to the higher salinity found in the eastern Mediterranean. Similarly, the less negative δ^13^C values exhibited by individuals from the Gulf of Cádiz are indicative of the coastal environment in which they inhabit, when compared to individuals from the pelagic environment of the Azores. Examination of a subset of the data that derives only from either skin or muscle samples (Figure 3B) appears to show no obvious dichotomy between tissue types, so inferences from the combined dataset are likely to be robust.

**FIGURE 4:**
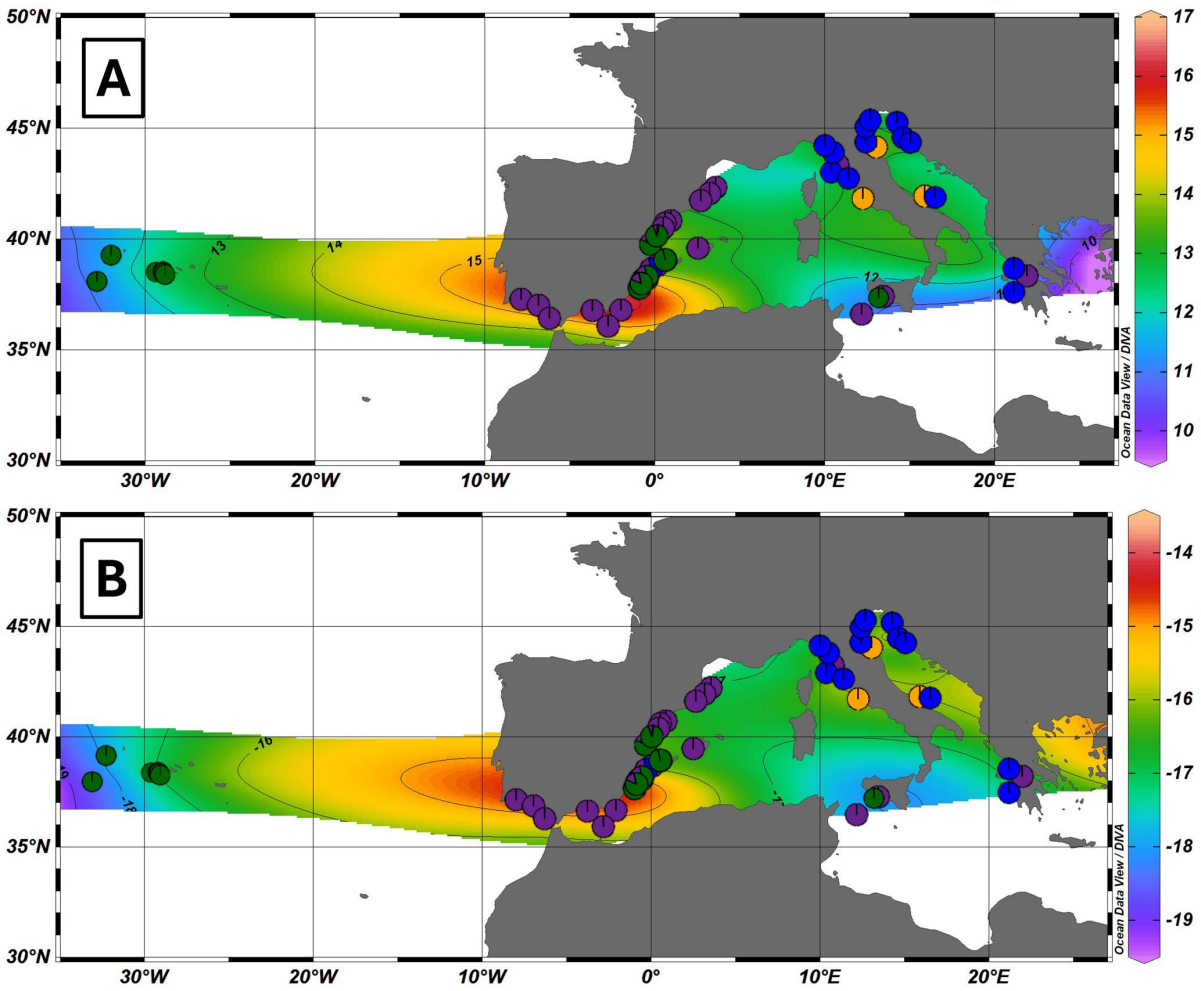
(A) Isoscape for δ^13^C generated from all samples of *T. truncatus* . Pie charts represent genetic structure as informed by Admixture. (B) Isoscape for δ^15^N generated from all samples of *T. truncatus* . Pie charts represent genetic structure as informed by Admixture. Isoscapes plotted using Ocean Data View.

The δ^13^C isoscape (Figure 4A) depicts the classic understanding of higher values in coastal waters and lower values in pelagic habitats. All coastal waters had a δ^13^C value near −16‰, whereas some pelagic environments were approximately −18‰. Particularly high δ^13^C values (−14.7‰), indicating enrichment of ^13^C, were observed around the coast of Spain, especially the north coast of the Alborán Sea. Coastal waters of the East Adriatic also had high values (≈−15.5‰). The isoscape for δ^15^N (Figure 4B) revealed a similar visual pattern to that of δ^13^C (Figure 4A) and some distinctions, such as in the Adriatic region (though see Figure S12 where only skin samples were used). Higher δ^15^N values, indicative of greater enrichment for ^15^N and feeding at higher trophic levels, were seen in coastal waters throughout the study area. Again, the highest δ^15^N values were seen around the Iberian Peninsula, in particular Gulf of Cádiz and the north coast of the Alborán Sea. There were also elevated levels in the southern Adriatic compared to surrounding regions, with particularly high δ^15^N values (+14.5‰) seen between the Italian region of Puglia and the Greek coast, though sample sizes are small.

**FIGURE 5:**
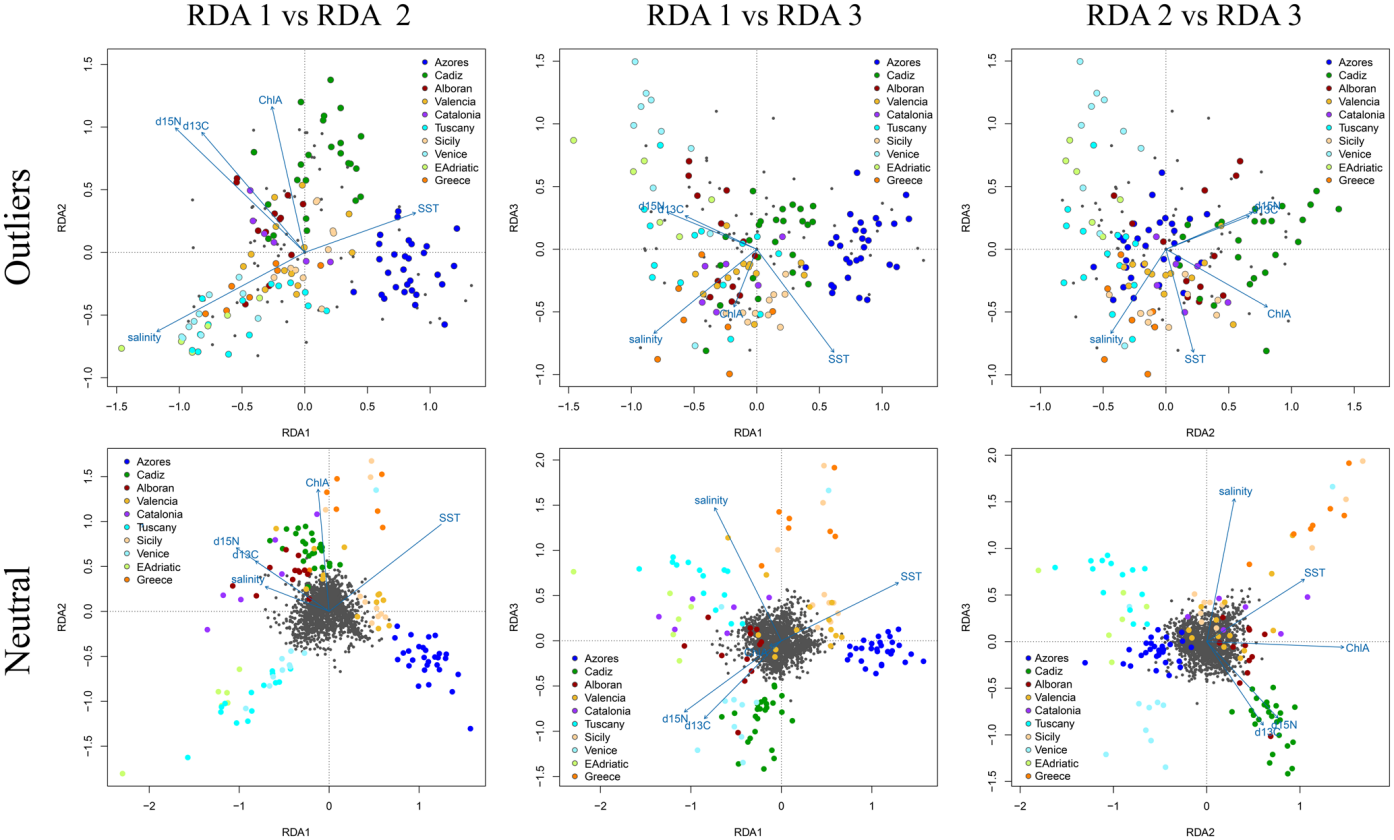
Redundancy Analysis (RDA) plots investigating the correlation between environmental variables and genetic variation. The lower row depicts neutral loci used to examine pure population structure whereas the upper row uses only outlier loci to investigate local adaptation. Sample regions are Azores (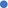), Cadiz (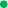), Alborán (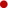), Valencia (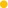), Catalonia (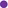), Tuscany (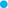), Sicily (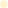), Venice (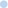), Eastern Adriatic (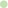) and Greece (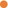).

Redundancy Analysis (RDA) (Figure 5) was based on 10 putative populations (excluding the Black Sea), extended to reflect physical differences in the environment. These putative populations were the Azores, Cadiz, Alborán, Valencia, Catalonia, Tuscany, Sicily, Venice, Eastern Adriatic and Greece. There were 138 samples with genetic data that passed quality filters and only 76 with SIA data, so for 62 samples the SIA values were represented by the regional mean values (defined by the available 76 samples). Details of *F*_ST_ values, sample sizes and average environmental values are given in Table S4. The sub‐populations were chosen such that all showed differences in either environmental, SI or genetic measures, but not all were significantly differentiated according to *F*_ST_ (Table S4). RDA was first carried out on neutral loci only. Overall, the constraining variables (environmental factors) explained 6.17% of the total genetic variance and four of the five tested factors were significant (*p* < 0.001). RDA1 was best represented by SST and explained the largest proportion of the constrained variance (28.53% of the 6.17%). RDA2 mostly reflected ChlA and explained 25.05% of the constrained variance. RDA3 mostly reflected salinity, and to some extent both δ^15^N/δ^13^C isotopes, explaining 19.59% of the constrained variance. Investigation of variance inflation in the *R* package *Vegan* indicated that no factors showed evidence of collinearity. RDA of outlier *loci* (Figure 5) explained a greater proportion of the total variance (10.25%). The representation of the different factors was somewhat similar with RDA1 = SST and RDA2 = ChlA; however, RDA3 was also best represented by SST and salinity, while δ^15^N was best represented in RDA2 (δ^13^C was non‐significant). RDA1 explained 43.87% of the constrained variance, RDA2 explained 26.44%, and RDA3 explained 14.33%. All were significant at *p* = 0.001, except RDA3 which was significant only at *p* = 0.05.

## 4 Discussion

This study set out to assess if marine environmental features drive the formation of population structure in a highly mobile apex marine predator. Natoli et al. (2005) identified a clear East–West divergence in the population structure of *T. truncatus* in the Mediterranean Sea, which included differentiation across the Siculo‐Tunisian Strait, a pattern observed in other large marine predators (Boustany et al. 2008; Carlsson et al. 2004; Gaspari et al. 2007; Natoli et al. 2008). Excluding samples from Sicily, we also found an East–West divergence in the Mediterranean but aligned further west than that found by Natoli et al. (2005), possibly along the longitudinal line formed by Corsica and Sardinia, though more samples are needed to confirm this. Division along the Corsica‐Sardinia line has been observed for other species in the Mediterranean (see Davies et al. 2011; De Innocentiis et al. 2004; Montes et al. 2012). Given that both movements and the distribution of *T. truncatus* have been shown to be strongly influenced by the distribution of prey species (Hastie et al. 2004), future studies of *T. truncatus* diet in this region would be useful for further investigation.

The Almería‐Oran front (AOF) has also been implicated in population structure formation in a number of marine predators (Bourret et al. 2007; Cimmaruta et al. 2005; Galarza et al. 2009; Schunter et al. 2011; Bearzi and Genov 2021). Our study revealed strong support (*Admixture* and *Barrier* analysis) for the AOF representing a population boundary in *T. truncatus* , with individuals found north along the Spanish coast (Figure S1) genetically differentiated from those found in the Alborán Sea. However, we lacked samples from the Costa de Almería region and in general more inclusive sampling across that region including the North African coast would help interpret the role of the AOF in restricting connectivity. The stable isotope data in this study reveal clear signals of differential feeding between these populations, with *T. truncatus* from the Gulf of Cádiz and Alborán Sea feeding at a higher trophic level than those off the coast of Valencia. In agreement with SIA data presented here, *T. truncatus* from the Gulf of Cádiz is known to feed on large demersal fish species such as European Hake *Merluccius merluccius* and European *Conger Conger conger* (Giménez et al. 2017). Although larval retention by the AOF alone is unlikely to be enough to create a barrier to gene flow in potential prey species (Naciri et al. 1999), the AOF's strong temperature differential, caused by the meeting of cold surface water from the Atlantic and warmer water flowing down from the Ibiza Channel, may be enough to influence prey species distribution and in so doing act as a potential mechanism for isolation of prey‐specialist *T. truncatus* populations.

This pattern of oceanic frontal regions or steep environmental clines influencing population structure has been suggested for a number of cetacean taxa worldwide (Fontaine et al. 2007; Fullard et al. 2000; Kasuya et al. 1988; Mendez et al. 2011) including in *Tursiops* spp. (Bilgmann et al. 2007; Natoli et al. 2005). All of these studies implicate the oceanographic and physiographic influence on prey distribution as being a likely key driver and the addition of stable isotope data in this study supports this interpretation.

In addition to genetic divergence across the AOF, this study also found a broader geographic correlation between genetic population structure of *T. truncatus* and ocean water environmental variables, as shown by significant RDA test results for both SST and salinity. Both are known to have a profound impact on the distribution of fish (Albert 2007; Castillo 1996; Sabatés et al. 2006), cephalopods (Fernández et al. 2011; Lansdell and Young 2007; Puerta et al. 2015) and crustaceans (Hall and Thatje 2009; O'Hara and Poore 2000), all of which are known to be prey species to a greater or lesser extent for different *T. truncatus* populations (Blanco et al. 2001; Giménez et al. 2017; Gladilina and Gol'din 2014; González et al. 1994; Santos et al. 2001). The RDA analysis also found a significant proportion of the genetic variance explained by ChlA and δ^15^N, which likely reflects trophic position. This is consistent with the patterns seen, especially the isotopic and genetic differentiation of the Cádiz region. The correlation between the distribution of cetaceans and ocean environmental features, driven by the physiological limits of prey species, is well documented (Selzer and Payne 1988; Tynan et al. 2005) and so it remains likely that prey distribution is a primary driver of genetic differentiation in *T. truncatus* in the Mediterranean, as has been suggested before for this species (Bilgmann et al. 2007; Fruet et al. 2017; Natoli et al. 2005; Sellas et al. 2005).

The Black Sea population of bottlenose dolphins has previously been identified as a genetically distinct population (Moura et al. 2020, 2013; Natoli et al. 2005), viewed as a subspecies of *T. truncatus* ( *T. truncatus ponticus* ; Viaud‐Martinez et al. 2008), and was somewhat differentiated in our study as well. Whilst *T. truncatus* spp. are seen within the Istanbul Strait (also known as the Bosporus; Bas et al. 2017), this body of water that separates the Black Sea from the Sea of Marmara is not only physically restricted (700 m wide at its narrowest) but is also one of the busiest shipping lanes in the world with high levels of anthropogenic disturbance, presenting a potential barrier to movement. *T. truncatus* spp. encounter rates are significantly higher in the adjacent seas (Akkaya Baş et al. 2019) and specialisation for prey species that may be geographically restricted by the lower salinity waters of the Black Sea, could also help maintain genetic separation between the Black Sea and Mediterranean populations. Analysis using *Barrier* in this study identified the largest barrier to gene flow as being between the Black Sea and all other regions, in agreement with previous studies.

Coastal *T. truncatus* in the northern Adriatic are relatively well studied and are known to exhibit fairly high site fidelity in several areas (Bearzi et al. 1997; Genov et al. 2008, 2019; Pleslić et al. 2015; Gaspari, Scheinin, et al. 2015). Relatively few individuals appear to move between study sites, thus potentially limiting gene flow (Genov et al. 2009; Pleslić et al. 2019), although some individuals have been documented moving substantial distances within the Adriatic Sea (Genov et al. 2016) and even extremely long distances across the Mediterranean Sea (Genov et al. 2022). We find an East–West separation for *T. truncatus* in the Adriatic consistent with Gaspari, Holcer, et al. (2015) who reported a similar potential split between East and West Adriatic bottlenose dolphins. The principal environmental difference between the east and west in the northern Adriatic is salinity. The Italian side of the basin is heavily influenced by the river Po, reducing the salinity levels of the waters along the coast north of the estuary and for a considerable distance to the south (Russo and Artegiani 1996). It is possible that local adaptation to this slightly fresher environment, or specialisation in feeding on locally adapted prey, could be enough to support the genetic separation observed (perhaps through natal philopatry).

Since the last glacial maximum (LGM) the Adriatic Sea has gradually pushed northwards as sea levels have risen, opening several new habitats in turn. Around 10,000 years ago the north‐western part of the Adriatic was a large barrier‐lagoon estuary system, much like the favoured foraging grounds of *T. truncatus* in South Carolina (Gubbins 2002; Pate and McFee 2012), whilst the modern island‐dominated coastal area of Croatia was still dry land (Trincardi et al. 1996). The present Balkan coastline only became flooded later; thus, it is possible that this temporal succession of habitats, and environmental differences between them, could have provided the context for the initial formation of the population structure observed in this study through a series of founder events. Similarly, Gaspari, Scheinin, et al. (2015) proposed that a series of founder events could be responsible for the observed population structure of *T. truncatus* in the wider Eastern Mediterranean region. However, further factors must be responsible for the contemporary maintenance of the observed population structure in the Adriatic, with prey and/or habitat specialisation, or social factors (Genov, Centrih, et al. 2019), being possible mechanisms.

A novel observation in our study was that *T. truncatus* from Sicily, Valencia and the Azores, although showing some inter‐population differentiation (see *F*_ST_ results, Table 1), consistently clustered together during analyses (Figure 1, Figure S6). Consistent with this, Sicilian *T. truncatus* have been shown to share acoustic characteristics in their vocalisations with their Macaronesian (Azores, Madeira and Canary Islands) counterparts (Papale et al. 2014) and there is greater acoustic similarity between these populations than any others that have been studied in the Mediterranean (La Manna et al. 2017).

Beyond acoustics there are further social and cultural similarities between the Sicilian and Macaronesia *T. truncatus* populations. Papale et al. (2017) conducted a study of social association patterns and site fidelity in the *T. truncatus* found off the south coast of Sicily and found that *T. truncatus* in this region had high social fluidity, with individuals associating in groups changing at relatively short timescales, and very low site fidelity (40% of dolphins sighted were only seen once and even those individuals that were deemed ‘more resident’ left the study area for significant periods of time). This is in contrast to populations observed elsewhere in the Mediterranean (Bearzi et al. 2008; Benmessaoud et al. 2013; Blasi and Boitani 2014; Díaz López and Shirai 2008; Genov et al. 2008, 2019; Gnone et al. 2011; Pleslić et al. 2015) and much more akin to those seen in Macaronesia (Dinis 2014; Dinis et al. 2021; Silva et al. 2008).

Investigations of diet using δ^13^C and δ^15^N indicated a strong coastal and high trophic level for all *T. truncatus* individuals from the Gulf of Cádiz. The high δ^13^C value for this local population can likely be attributed to a strongly associated coastal lifestyle (see Michener and Kaufman 2007) and it could be argued that these individuals fit the coastal ecotype better than the pelagic ecotype as suggested by Nykänen et al. (2019). In either case, their enrichment of δ^15^N, and presumed feeding at a higher trophic level, fits with their reported dietary preferences of large predatory fish species such as European Hake ( *Merluccius merluccius* ) and European Conger ( *Conger conger* ; Giménez et al. 2017). These findings contrast strongly with those of the putative Azores–Sicily metapopulation, which had lower δ^13^C and δ^15^N values. The difference in pelagic vs coastal δ^13^C has been used previously to differentiate between the two *T. truncatus* ecotypes, with offshore ecotypes usually being slightly depleted for δ^13^C (Barros et al. 2010), thus adding further evidence that Azorean, and possibly Sicilian, *T. truncatus* are of the offshore ecotype (Quérouil et al. 2007).

Investigations into the historical demography of *T. truncatus* populations revealed a general trend of population decline over the past 100,000 years (with the exception of Atlantic and Greek populations which remained stable over this period). Some caution should be applied in the interpretation of these trends as many methods for estimating population historical demographics assume a model of a single panmictic population (Chikhi et al. 2018; Mazet et al. 2016). Effects of violations of this assumption can include false signals of population decline (Heller et al. 2013), although SFS methodologies, as used in this study, are known to be more resilient (Excoffier et al. 2013; Lohmueller et al. 2010). Nearly all population declines displayed a distinct stepped pattern, suggestive of potential threshold tipping points resulting from environmental changes. However, apart from the Sicilian population (which showed dramatic population declines following the onset of the last glacial period and the last glacial maximum) the periods of steepest decline do not correspond with any of the major geologic or environmental events investigated. It is possible that recent admixture may be influencing either the timing or magnitude of these events (Lohmueller et al. 2010). An interesting observation from the historical demographies is that the population trends of Valencia, Alborán and Cádiz are nearly identical. This could suggest they have all been influenced by some environmental factor, or admixture event, that is specific to the Iberian Peninsula, despite being genetically differentiated.

In this study we show that a highly mobile apex marine predator shows population genetic structure associated with both environmental factors and apparent prey choice. The data further suggest that the offshore ecotype found in the open Atlantic is found deep within the Mediterranean Sea, clustering with respect to both genotypes and prey choice. Differentiating between direct physiological adaptation to environmental factors and indirect trophic effects can be difficult, with both known to be a pathway for the formation of population structure in Cetacea (Pratt et al. 2022). However, this study presents evidence that prey choice is a major evolutionary driver within cetaceans, and likely reinforced by cultural learning and social structure, a notion supported by a study of social partitioning in one of the areas within our study region (Genov, Centrih, et al. 2019). Prey specialisation has been shown to correlate with population structure for a number of highly mobile and broad‐ranging marine predators including other cetaceans (Moura et al. 2014; Méndez‐Fernandez et al. 2020; Buss 2022), pinnipeds (Lowther et al. 2012) and seabirds (Wiley et al. 2012). This makes it likely that, at least for endothermic predators capable of thriving in a range of environmental conditions, indirect trophic effects of the environment are a key driver of population structure formation.

## Author Contributions

D.M.M. and A.R.H. designed the study. D.M.M., A.N., E.P., E.G.C., M.A.S., T.G. and S.G. collected samples and data. D.M.M., A.E.M., A.R.H. and D.R.G. analyzed the data. A.R.H., A.N., A.E.M., G.B., P.B. and D.R.G. provided supervision and study steerage. D.M.M., A.R.H., A.E.M., E.G.C. and D.R.G. wrote this manuscript with edits from all other authors.

## Conflicts of Interest

The authors declare no conflicts of interest.

## Supporting information


**Data S1:** mec70182‐sup‐0001‐DataS1.pdf.

## Data Availability

Sequence and genotype data are available through Dryad at DOI: https://doi.org/10.5061/dryad.xsj3tx9vd. The code used in analysis is available on Zenodo at: https://doi.org/10.5281/zenodo.17560246. There are no restrictions on data availability.
