# Supplementary material for: Population Genomics and the Environmental Drivers of Population Structure in a Cosmopolitan Marine Predator, Tursiops truncatus
Source: Mol Ecol. 2025 Dec 1;34(24):e70182. doi: 10.1111/mec.70182 (PMC12717980; doi:10.1111/mec.70182)
Supplement: Supplementary file 1 — Data S1: mec70182‐sup‐0001‐DataS1.pdf. [file MEC-34-e70182-s001.pdf]

MOLECULAR ECOLOGY

Supplemental Information for:

Population genomics and the drivers of population structure in a cosmopolitan marine predator, *Tursiops truncatus*

Daniel M. Moore, Andre E. Moura, Ada Natoli, Elena Papale, Emily G. Cunningham, Mónica A. Silva, Tilen Genov, Stefania Gaspari, Giusi Buscaino, Per Berggren, Darren R. Gröcke, A. Rus Hoelzel

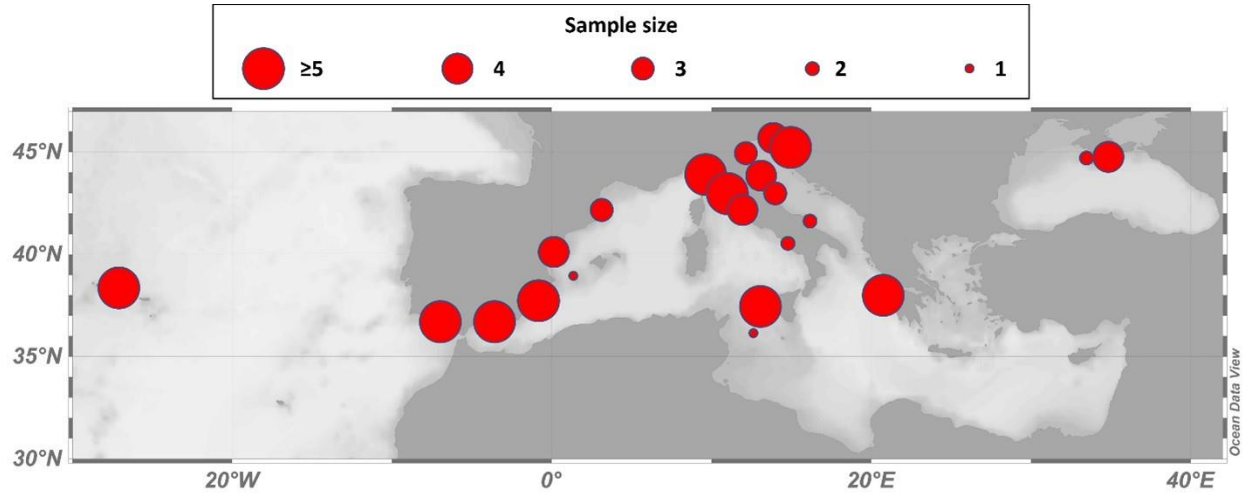

Figure S1: Geographic distribution of *Tursiops truncatus* genetic samples used in this study.

MOLECULAR ECOLOGY

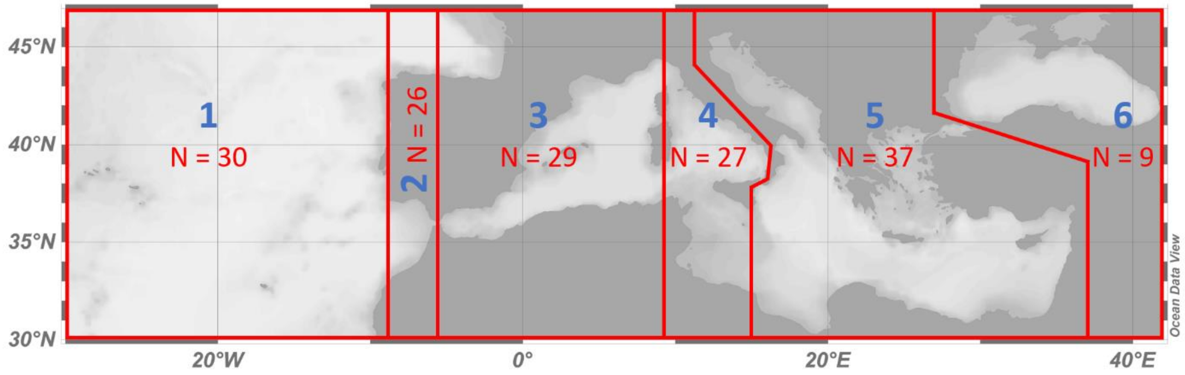

Figure S2: Schematic of population map as inputted into the Stacks v1.35 ref_map.pl pipeline based on geographic location and Natoli *et al.* (2005). Population names used in downstream analyses are 1:Azores, 2:Cadiz, 3:WestMed, 4:CentralMed, 5:EastMed and 6:BlackSea.

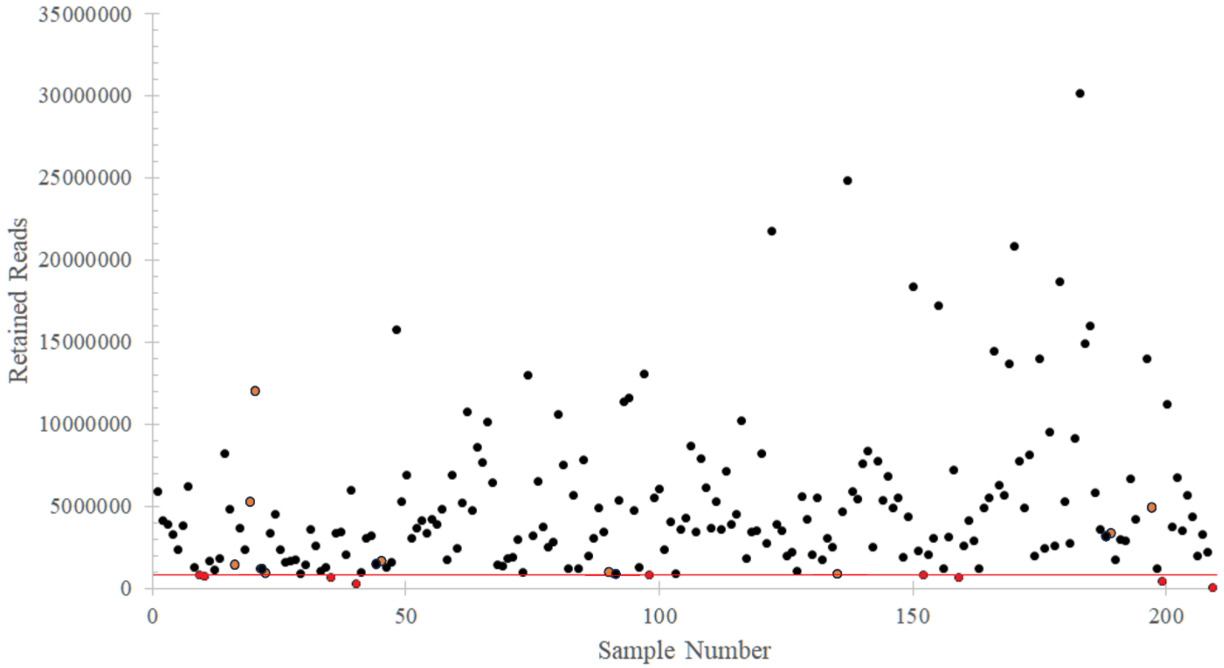

Figure S3: Number of retained reads per sample. Samples with less than 900,000 reads (highlighted in red, *n*=10) were removed from further analysis. Similarly, samples with greater than 30% missing data were also removed from further analysis (highlighted in orange, *n*=9).

MOLECULAR ECOLOGY

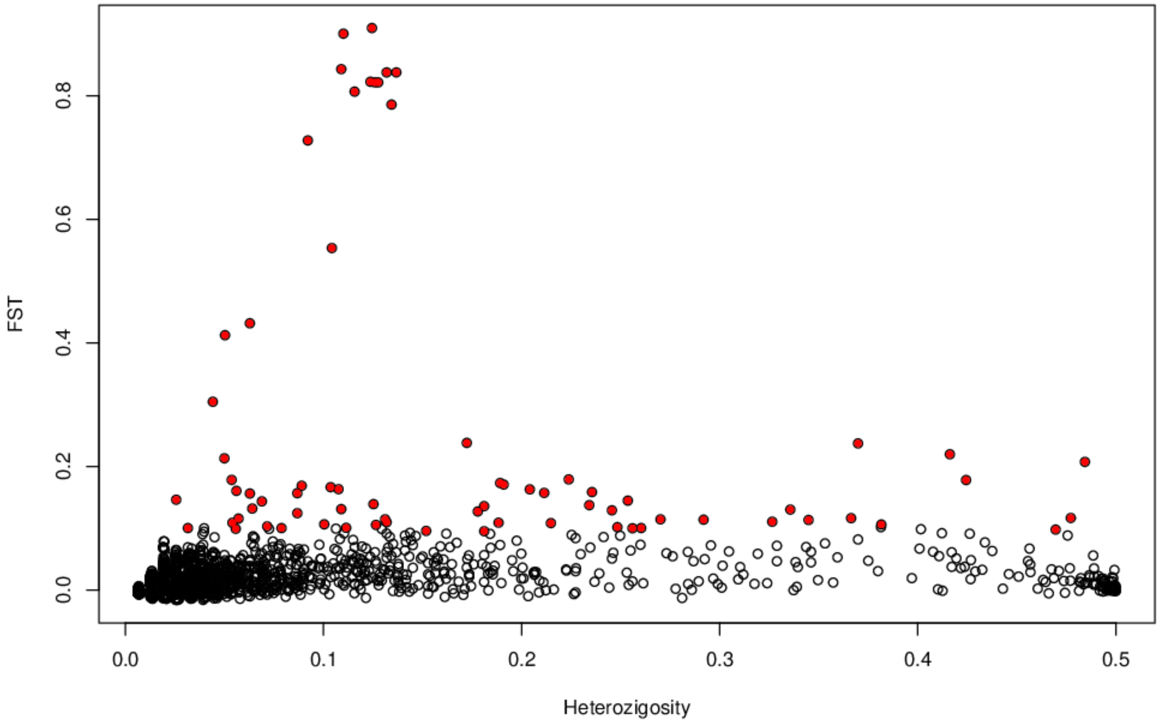

Figure S4: Detecting loci putatively under selection using the programs Lositan and Outflank. Loci highlighted in red (*n*=72) are outliers and putatively under positive selection. Loci in black (*n*=2641) are considered neutral.

MOLECULAR ECOLOGY

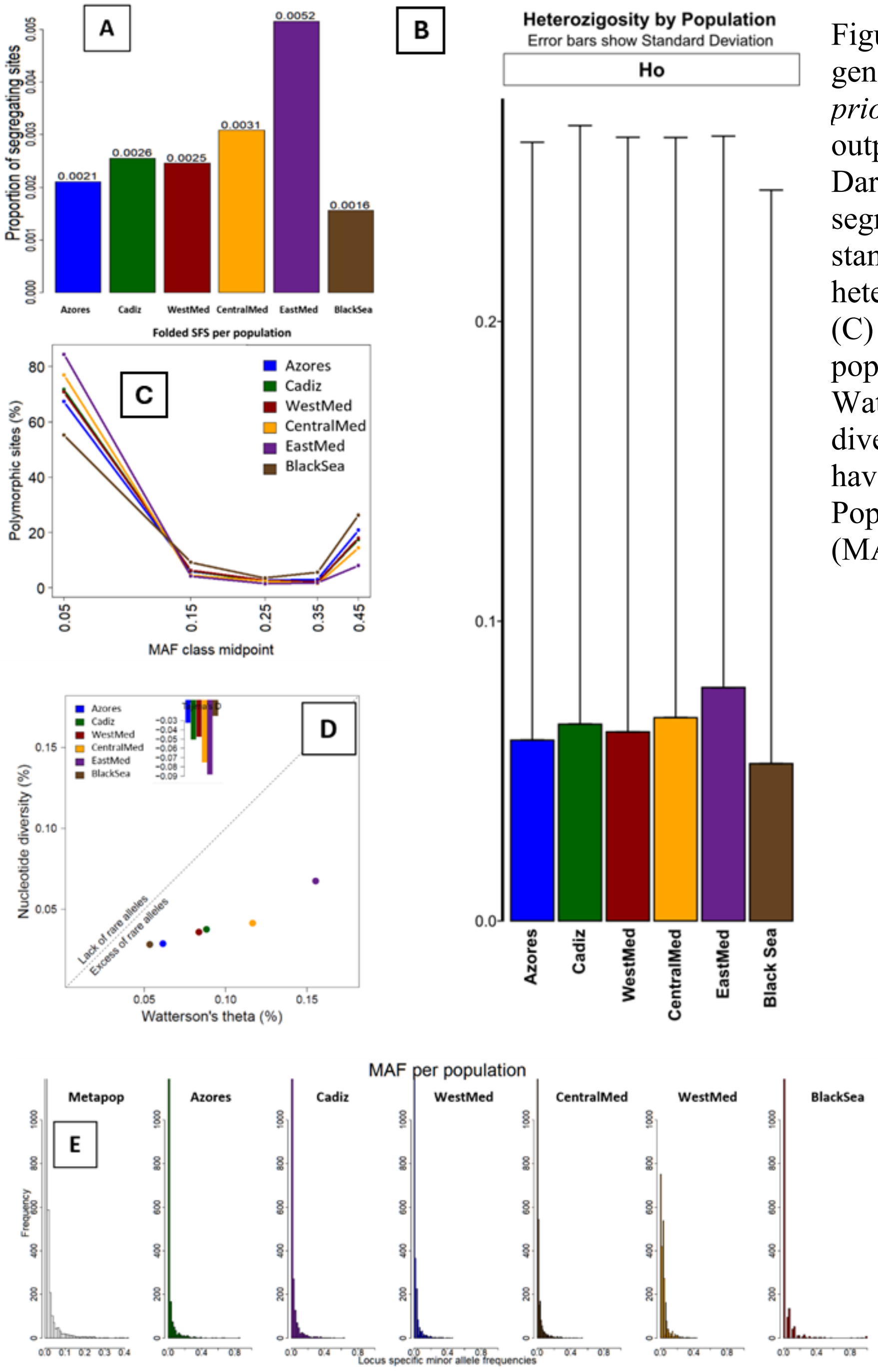

Figure S5: Investigations of genetic diversity between broad *a priori* populations. Analysis and output created in R (SambaR or DartR). (A) proportion of segregating sites (B) mean and standard deviation of heterozygosity for each population (C) allele frequency spectrum per population (D) comparison of Watterson's θ to nucleotide diversity (shows all populations have an excess of rare alleles) (E) Population Minor Allele Frequency (MAF)

.

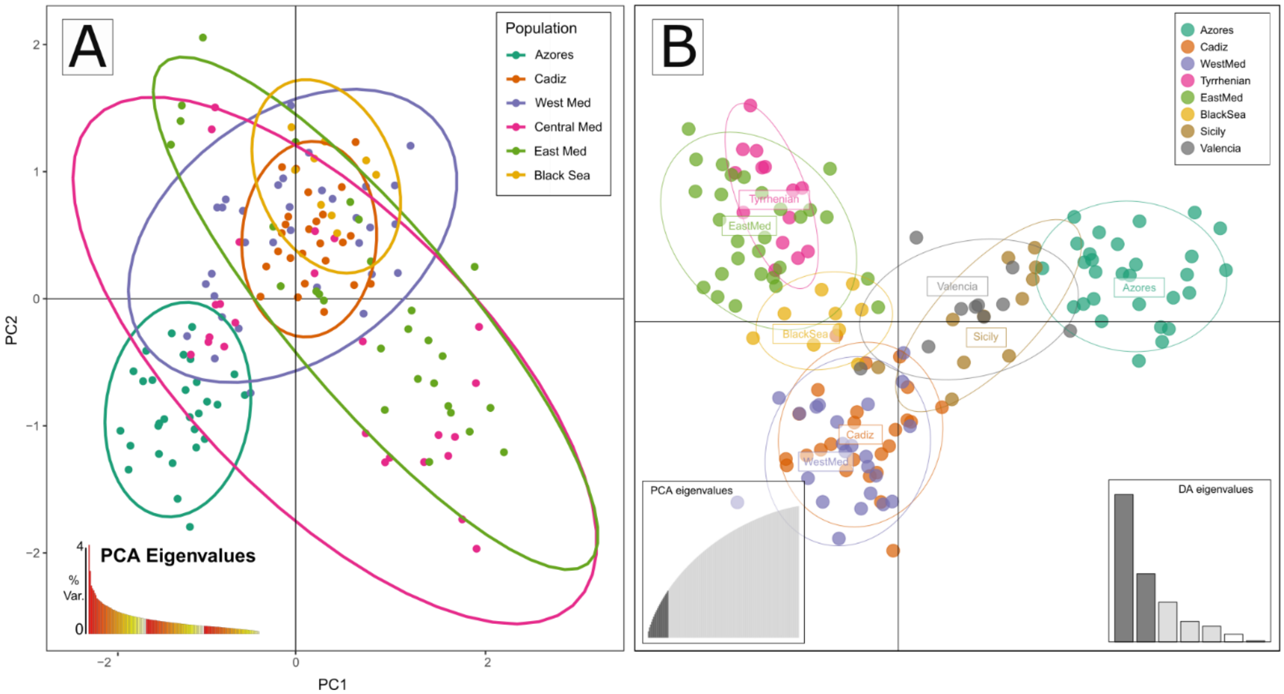

Figure S6: (A) Principal component analysis of samples based on neutral loci identified by Lositan and outFLANK. (B) Discriminant Analysis of Principal Components based on neutral loci as identified by Lositan and outFLANK clearly showing the clustering of Azores, Valencia and Sicily individuals.

MOLECULAR ECOLOGY

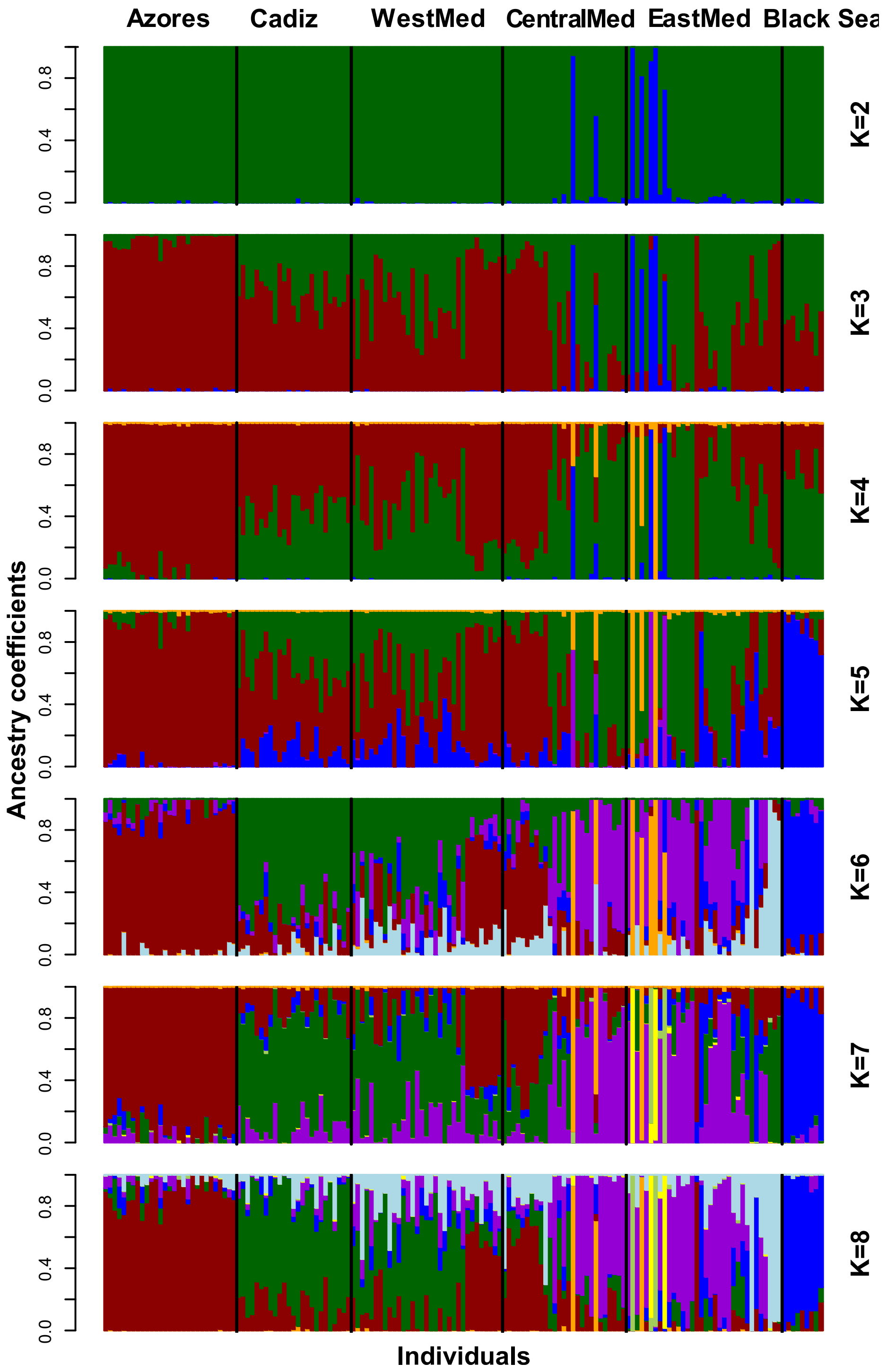

Figure S7: Estimated admixture coefficient for individuals across all estimates of K from 2-8. Plot derived from the snmf function in the *R* package LEA

MOLECULAR ECOLOGY

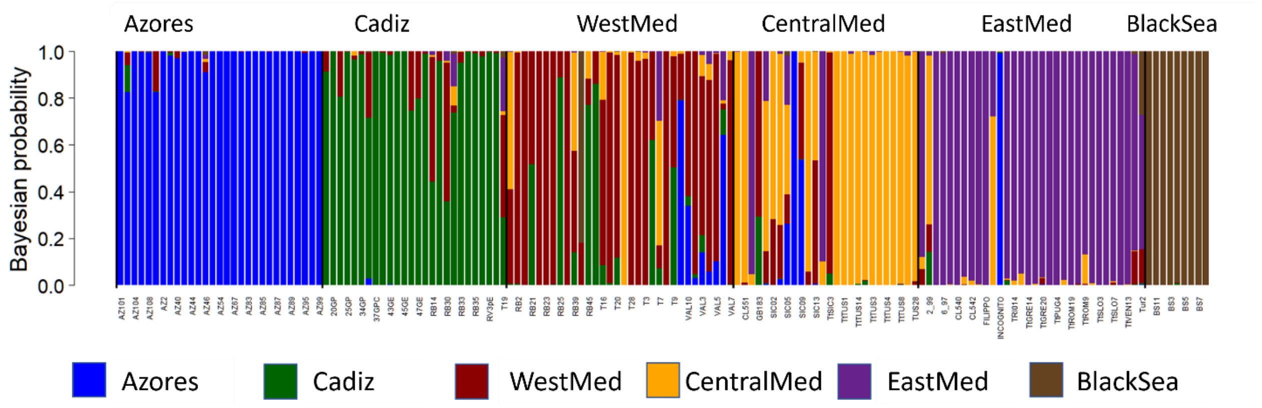

Figure S8: Bayesian likelihood barplot showing the probability that an individual belongs to a certain population given priors in Minor Allele Frequency. Estimations performed in SambaR.

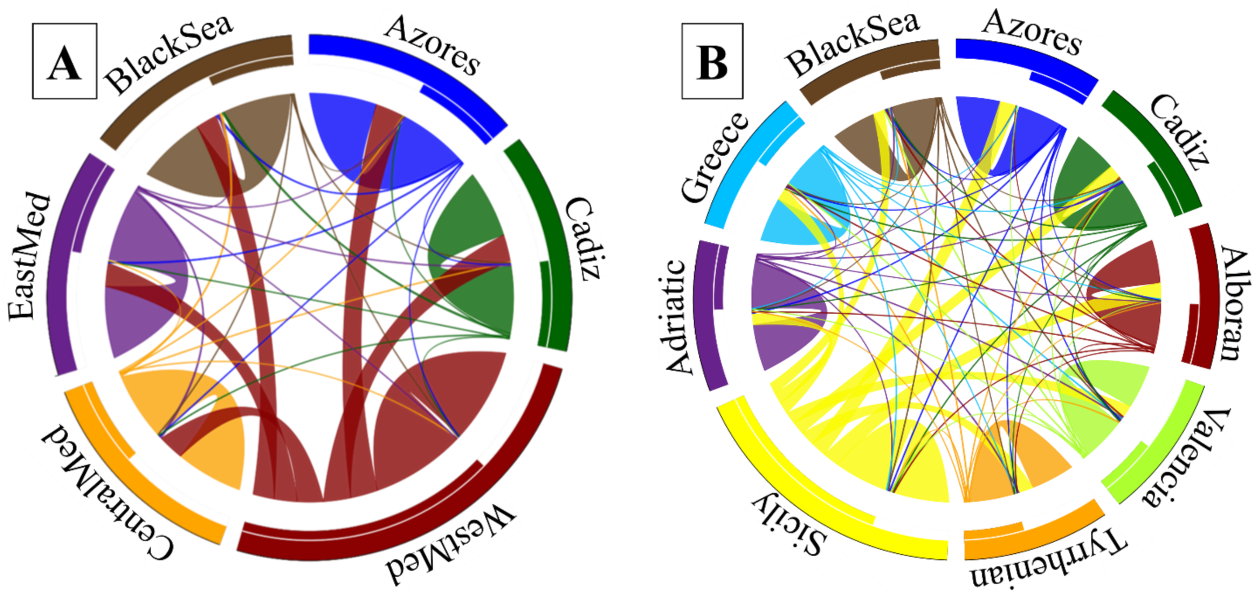

Figure S9: Circos plots showing migration rates between populations as calculated by BayesAss3-SNPs. A) shows *a priori* populations as defined in figure S2, with high levels of gene flow from WestMed to other areas. B) shows more locally defined populations with high levels of gene flow from Sicily.

A)

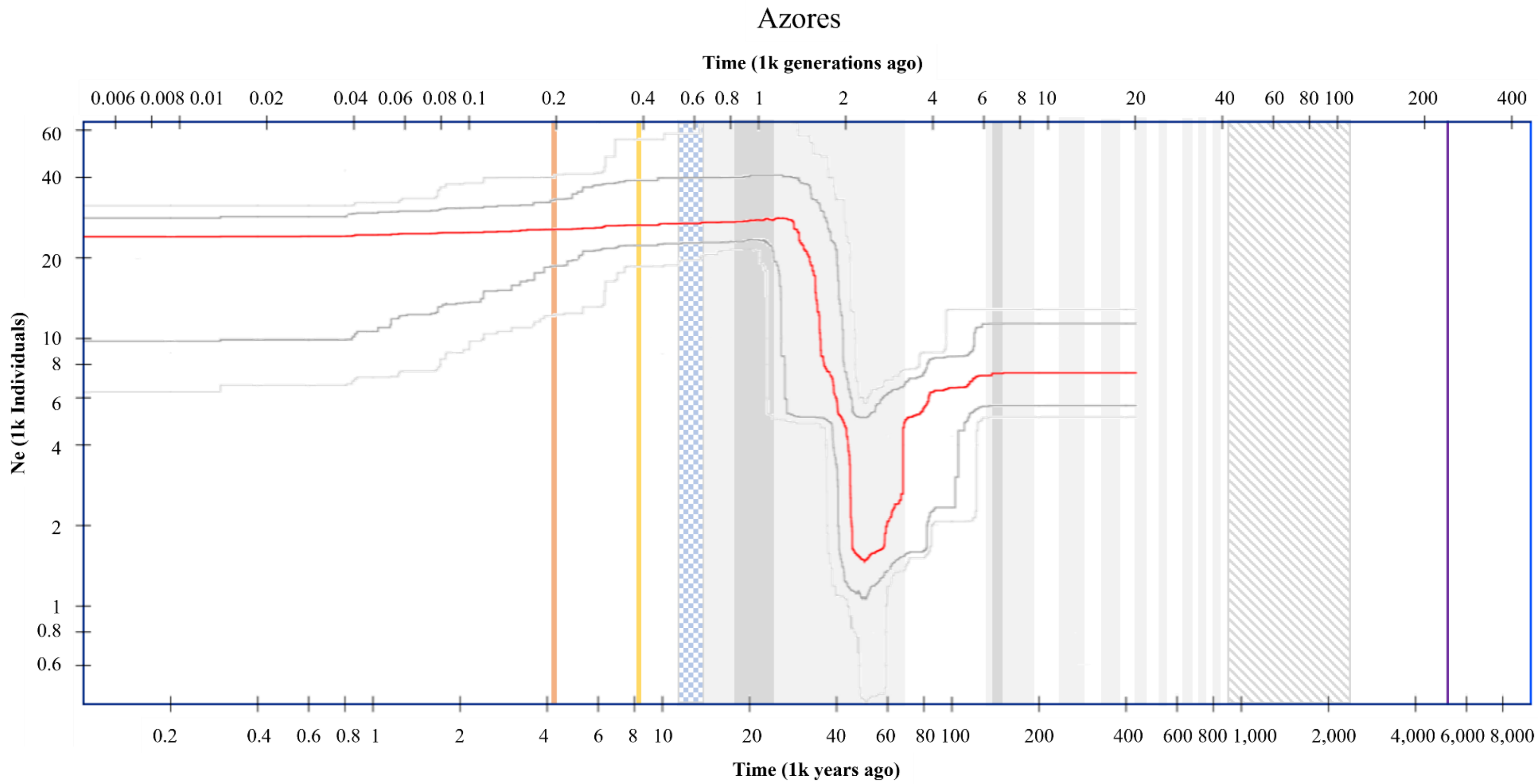
Azores
Time (1k generations ago)
0.006 0.008 0.01 0.02 0.04 0.06 0.08 0.1 0.2 0.4 0.6 0.8 1 2 4 6 8 10 20 40 60 80 100 200 400
Ne (1k Individuals)
60 40 20 10 8 6 4 2 1 0.8 0.6
0.2 0.4 0.6 0.8 1 2 4 6 8 10 20 40 60 80 100 200 400 600 800 1,000 2,000 4,000 6,000 8,000
Time (1k years ago)

B)

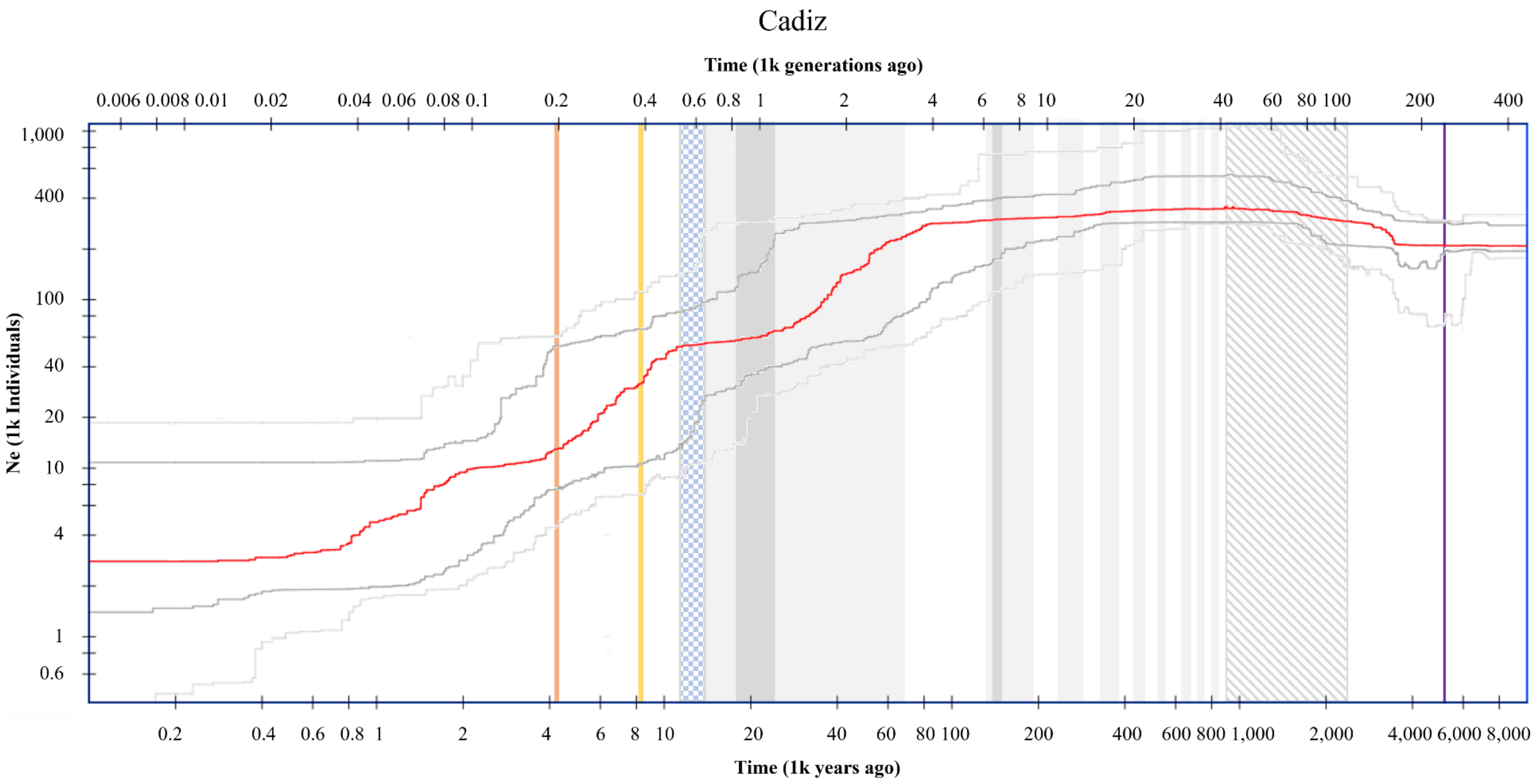
Cadiz
Time (1k generations ago)
0.006 0.008 0.01 0.02 0.04 0.06 0.08 0.1 0.2 0.4 0.6 0.8 1 2 4 6 8 10 20 40 60 80 100 200 400
Ne (1k Individuals)
1,000 400 100 40 20 10 4 1 0.6
0.2 0.4 0.6 0.8 1 2 4 6 8 10 20 40 60 80 100 200 400 600 800 1,000 2,000 4,000 6,000 8,000
Time (1k years ago)

MOLECULAR ECOLOGY

C)

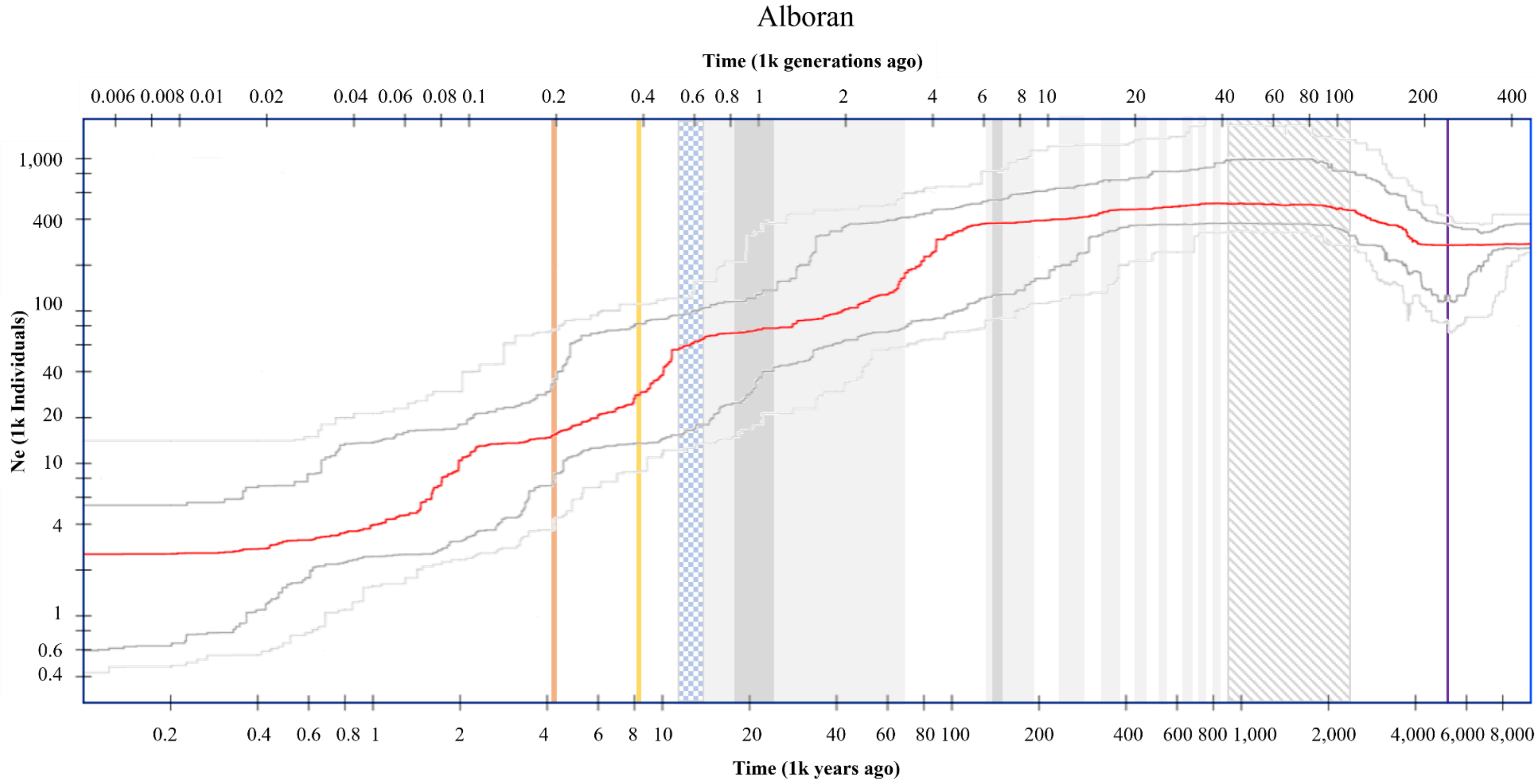

D)

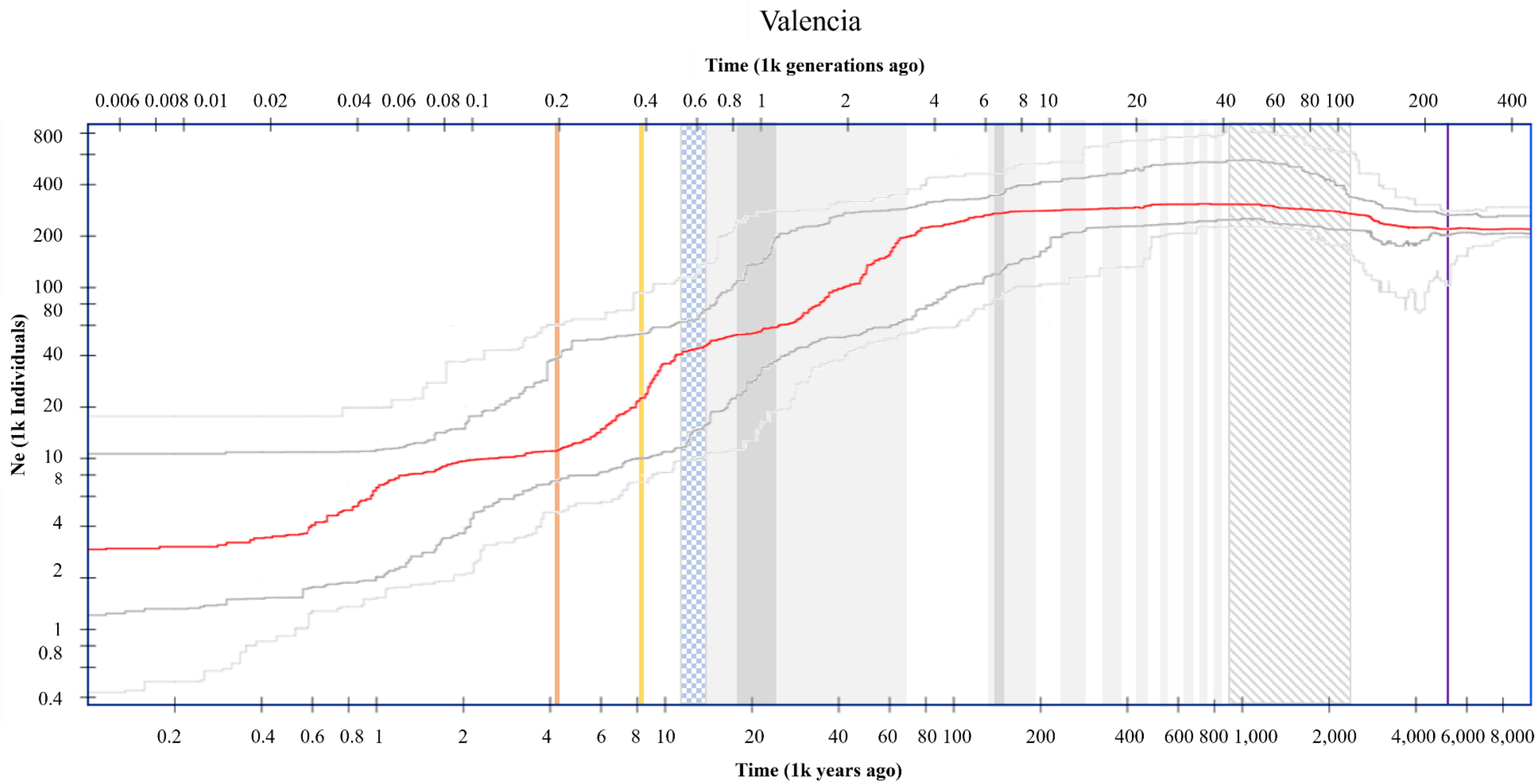
Valencia
Time (1k generations ago)
0.006 0.008 0.01 0.02 0.04 0.06 0.08 0.1 0.2 0.4 0.6 0.8 1 2 4 6 8 10 20 40 60 80 100 200 400
Ne (1k Individuals)
800 400 200 100 80 40 20 10 8 4 2 1 0.8 0.4
0.2 0.4 0.6 0.8 1 2 4 6 8 10 20 40 60 80 100 200 400 600 800 1,000 2,000 4,000 6,000 8,000
Time (1k years ago)

MOLECULAR ECOLOGY

E)

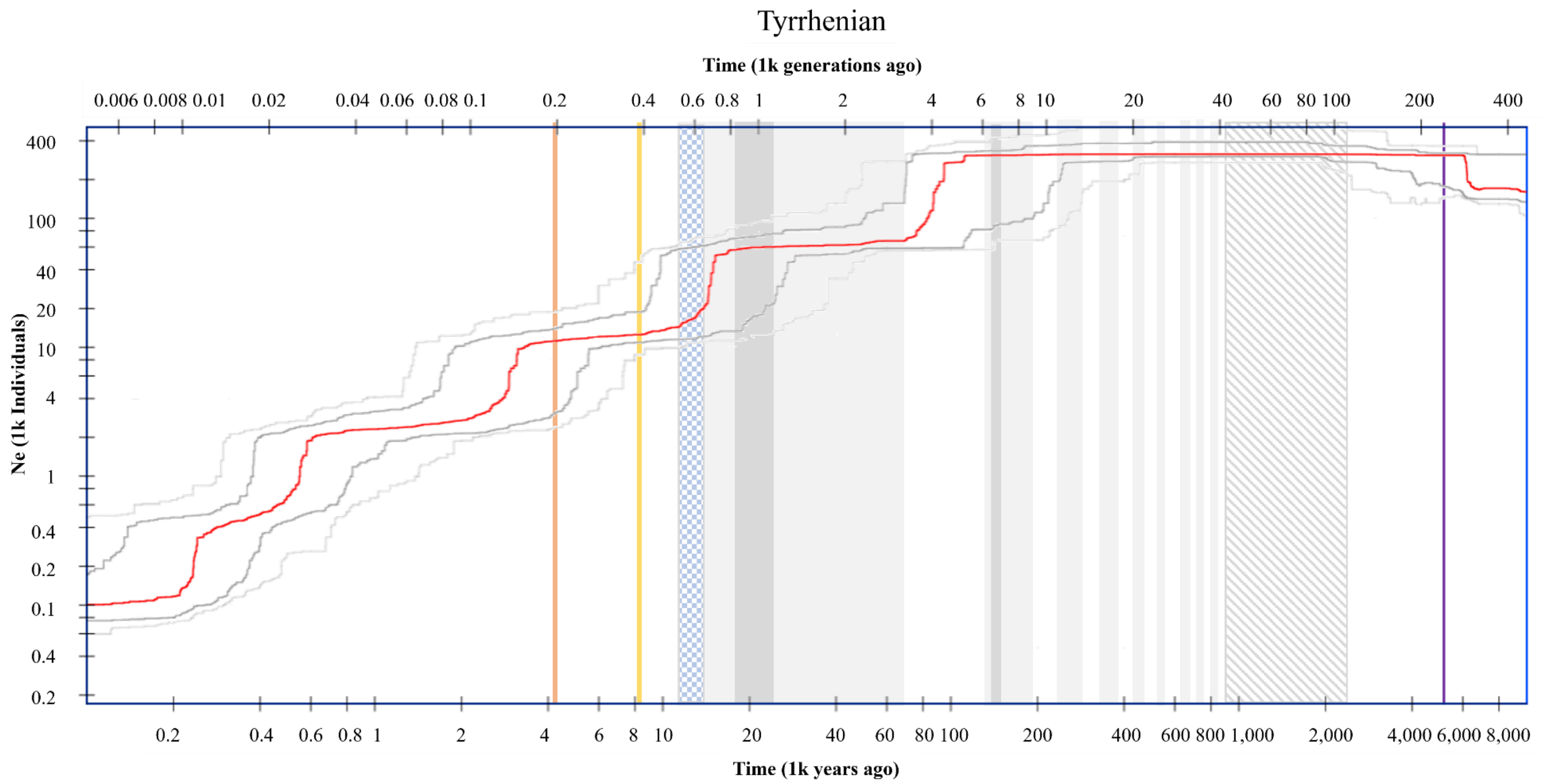

MOLECULAR ECOLOGY

F)

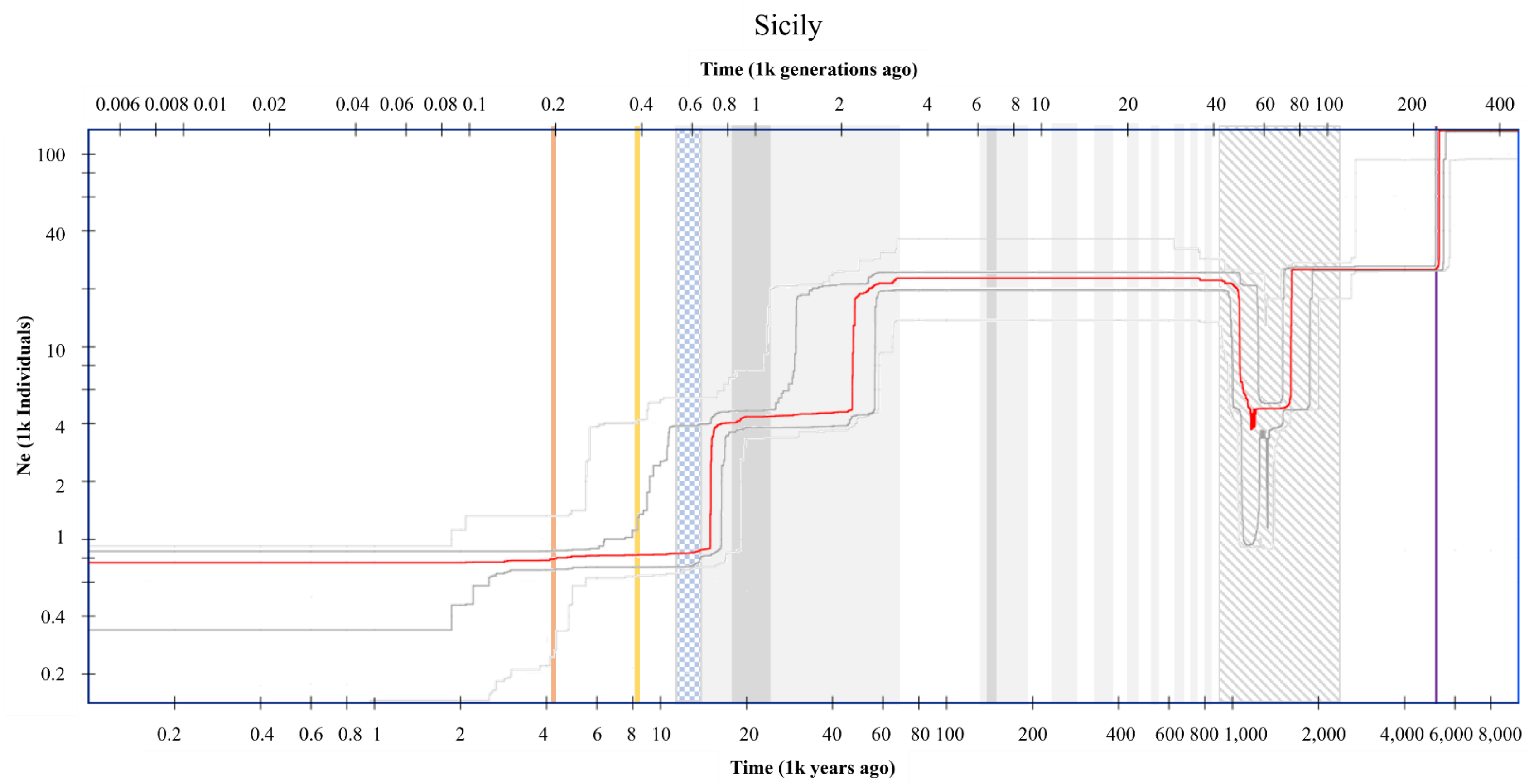

MOLECULAR ECOLOGY

G)

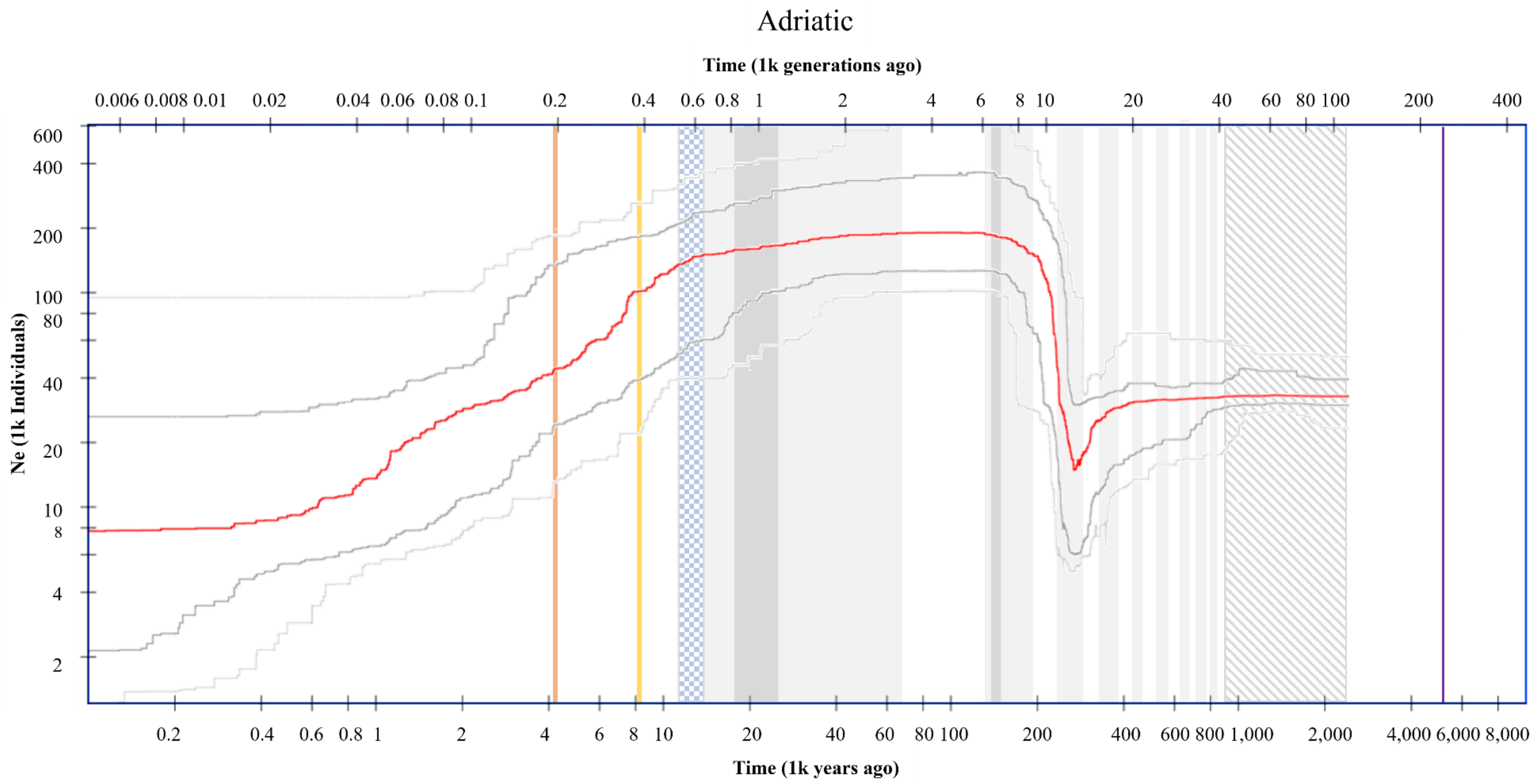

MOLECULAR ECOLOGY

H)

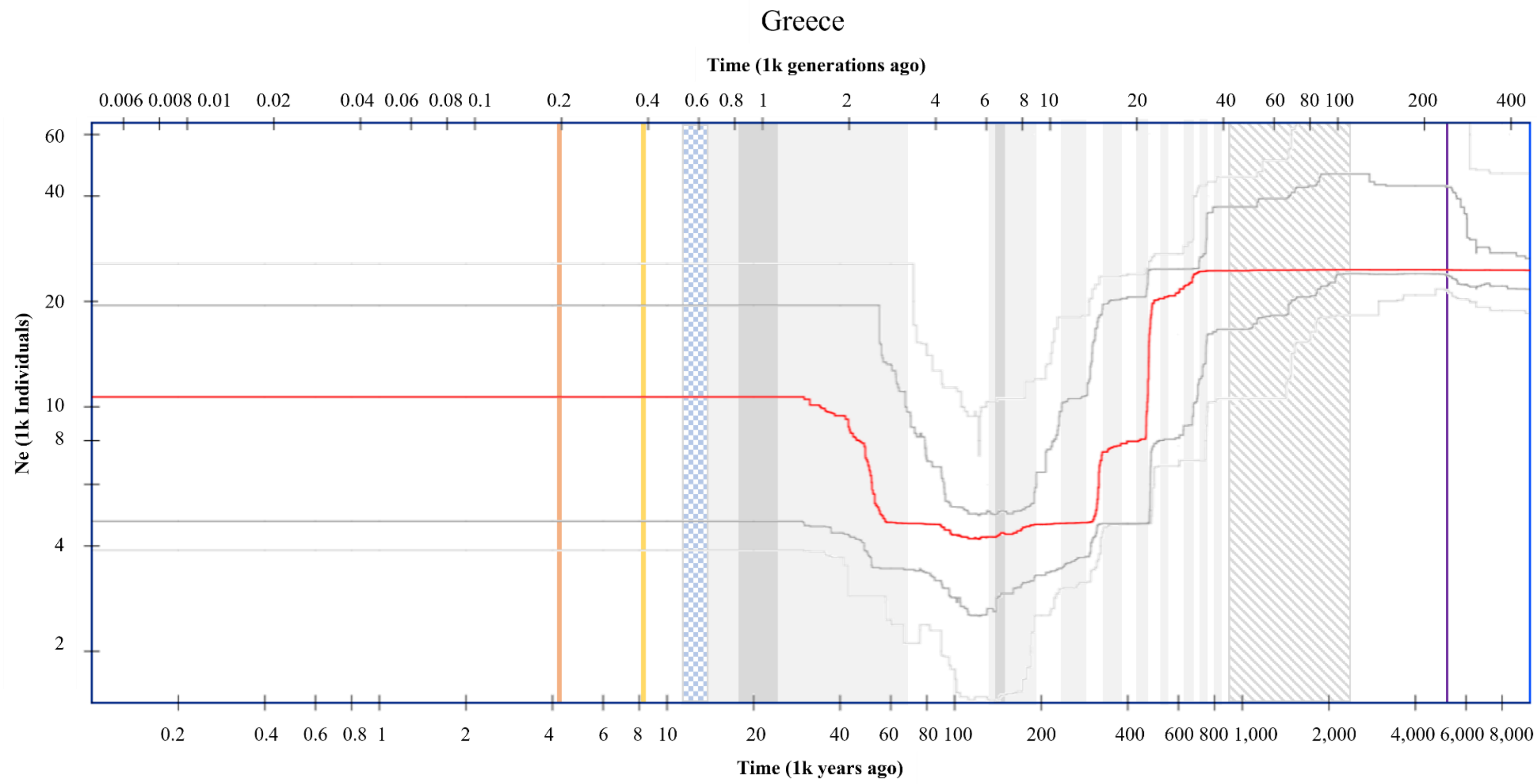

MOLECULAR ECOLOGY

I)

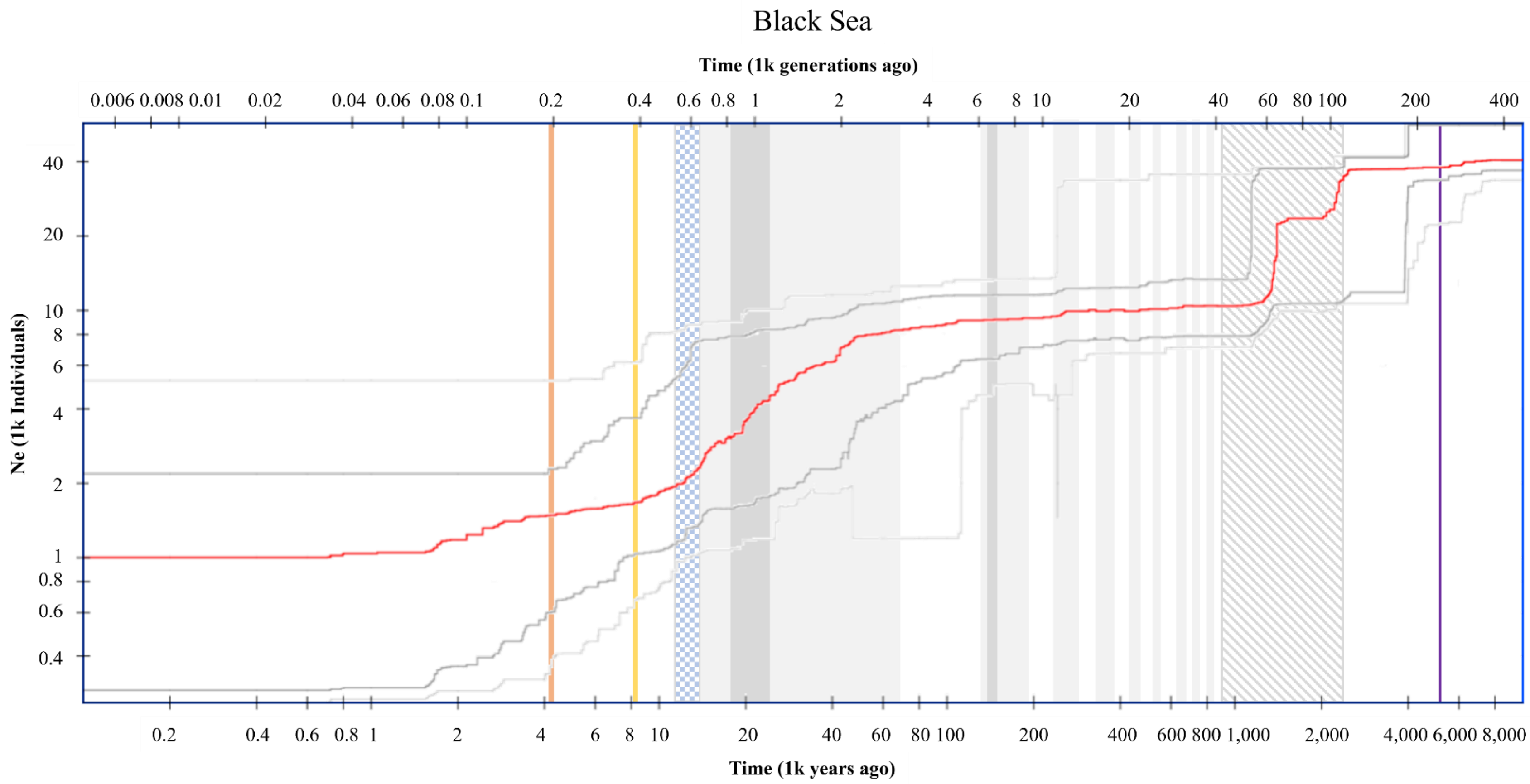

MOLECULAR ECOLOGY

Figure S10: Demography of the a) Azores, b) Cadiz, c) Alboran, d) Valencian, e) Tyrrhenian, f) Sicilian, g) Adriatic, h) Greek, and i) Black Sea populations as derived from ANGSD calculated SFS and annotated with various climate events of the past8 million years. Vertical grey bars indicate glacial periods as derived from temperature records of the the EPICA Dome C ice core (Masson-Delmotte *et al*., 2010) (dark grey vertical bars indicate the Last Glacial Maximum and the Penultimate Glacial Maximum). The glacial cycles of the Gelasian and early Calabrian are too fine to show on this scale and so this whole period is illustrated by the vertical block with diagonal lines. The Younger-Dryas event is indicated by the blue and white chequered vertical bar. The 4.2 kiloyear event, 8.2 kiloyear event and Zanclean flood are indicated by the orange, yellow and purple vertical lines respectively.

MOLECULAR ECOLOGY

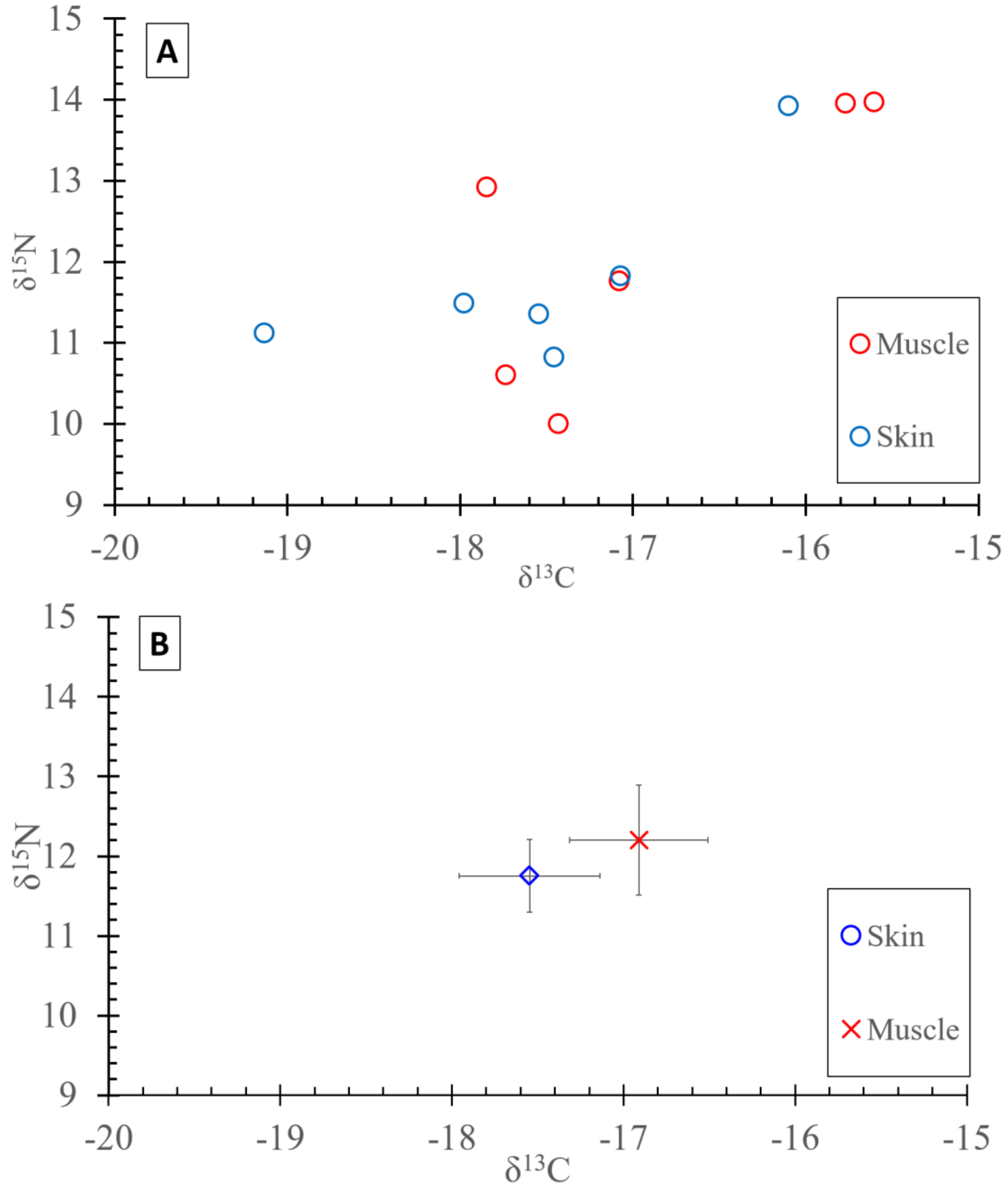

Figure S11: Stable isotope samples taken from the same location (Azores) were compared between two tissue types (skin, *n*=6 and muscle, *n*=6). $\delta^{13}C$ and $\delta^{15}N$ values for individual samples are shown in A and tissue means in B. Error bars are equal to one standard error.

MOLECULAR ECOLOGY

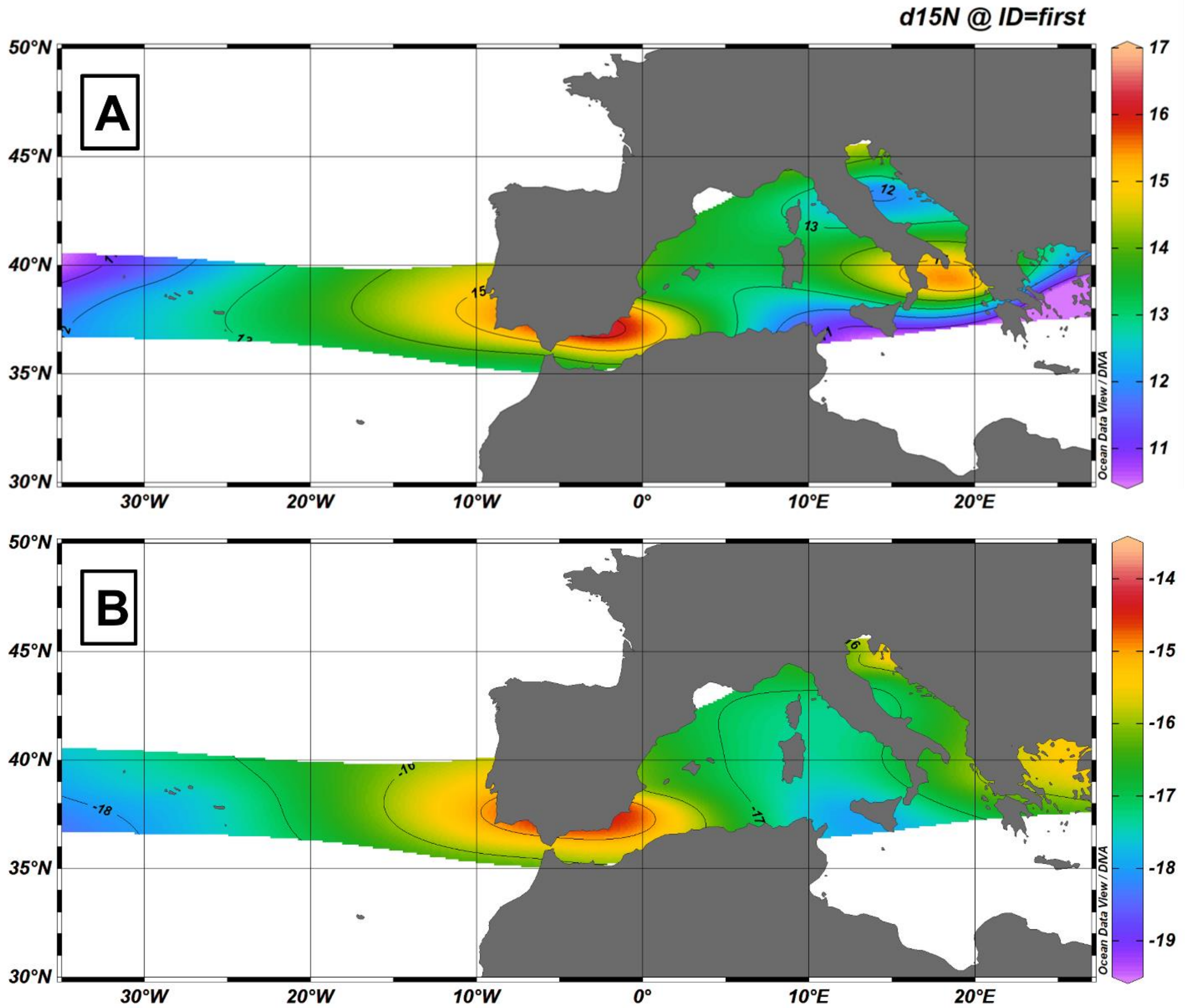

Figure S12: (A) Isoscape for $\delta^{13}C$ generated from skin only samples of *Tursiops truncatus*. (B) Isoscape for $\delta^{15}N$ generated from skin only samples of *T. truncatus*. Isoscapes plotted using Ocean Data View.

Table S1: Inferred posterior mean migration rates as calculated in BayesAss3-SNPs shown in Figure S9A. Migration rates (bold) can be interpreted as fraction of individuals in row population that are migrants derived from column population. Values underneath represents 95% CI set when ± to mean value.

	Azores	Cadiz	WestMed	CentralMed	EastMed	BlackSea
Azores	**0.6834**	**0.0096**	**0.2784**	**0.0096**	**0.0093**	**0.0096**
±	0.0302	0.0092	0.0346	0.0091	0.0092	0.0093
Cadiz	**0.0106**	**0.6813**	**0.2762**	**0.0107**	**0.0105**	**0.0107**
±	0.0100	0.0167	0.0244	0.0104	0.0103	0.0102
WestMed	**0.0087**	**0.0089**	**0.9569**	**0.0085**	**0.0084**	**0.0086**
±	0.0085	0.0086	0.0178	0.0083	0.0083	0.0084
CentralMed	**0.0106**	**0.0101**	**0.2619**	**0.6845**	**0.0227**	**0.0101**
±	0.0104	0.0100	0.0240	0.0139	0.0151	0.0099
EastMed	**0.0154**	**0.0084**	**0.2339**	**0.0083**	**0.7259**	**0.0081**
±	0.0115	0.0083	0.0279	0.0083	0.0254	0.0078
BlackSea	**0.0222**	**0.0220**	**0.2169**	**0.0221**	**0.0222**	**0.6947**
±	0.0208	0.0205	0.0449	0.0210	0.0206	0.0313

Table S2: Inferred posterior mean migration rates as calculated in BayesAss3-SNPs shown in Figure S9B. Migration rates (bold) can be interpreted as fraction of individuals in row population that are migrants derived from column population. Values underneath represents 95% CI set when ± to mean value.

	Azores	Cadiz	Alboran	Valencia	Tyrrhenian	Sicily	Adriatic	BlackSea
Azores	**0.6995**	**0.0094**	**0.0089**	**0.0092**	**0.0092**	**0.2457**	**0.0092**	**0.009**
±	0.0639	0.0092	0.0086	0.0089	0.0088	0.0656	0.0093	0.0085
Cadiz	**0.0157**	**0.6882**	**0.016**	**0.0151**	**0.0153**	**0.219**	**0.015**	**0.0157**
±	0.0148	0.023	0.0153	0.0146	0.0148	0.0365	0.0145	0.0146
Alboran	**0.0098**	**0.0097**	**0.6813**	**0.0096**	**0.0096**	**0.2609**	**0.0094**	**0.0096**
±	0.0093	0.0093	0.0171	0.0091	0.0092	0.0266	0.0091	0.0094
Valencia	**0.0181**	**0.019**	**0.0191**	**0.6849**	**0.0187**	**0.2038**	**0.0181**	**0.0183**
±	0.0175	0.0182	0.018	0.0172	0.0173	0.037	0.0173	0.0175
Tyrrhenian	**0.0138**	**0.0106**	**0.0138**	**0.0105**	**0.6882**	**0.2311**	**0.0213**	**0.0107**
±	0.0157	0.0101	0.013	0.0105	0.0147	0.032	0.0144	0.0102
Sicily	**0.0173**	**0.018**	**0.0175**	**0.0179**	**0.0173**	**0.8769**	**0.0173**	**0.0177**
±	0.0165	0.017	0.0166	0.0171	0.0165	0.0364	0.0165	0.017
Adriatic	**0.0147**	**0.0077**	**0.0078**	**0.0079**	**0.008**	**0.2155**	**0.7307**	**0.0077**
±	0.0112	0.0075	0.0076	0.0078	0.0079	0.0248	0.021	0.0077
BlackSea	**0.0288**	**0.0192**	**0.0202**	**0.0196**	**0.0195**	**0.1796**	**0.0191**	**0.694**
±	0.0361	0.0186	0.0189	0.0184	0.0185	0.0633	0.018	0.0317

Table S3: Stable Isotope data for 76 individuals used to create the ODV Isoscape maps (Figures 4 & S12)

**ID**	**δ^{15}N**	**δ^{13}C**	**Sea**	**Long**	**Lat**	**Tissue**	**Storage**
CL541-LE	13.88	-15.67	Adriatic	12.3676	44.24975	Skin	Frozen
CL546-LE	13.83	-16.46	Adriatic	12.3063	44.4598	Skin	Frozen
CRO101-LE	13.56	-14.17	Adriatic	14.52661	44.61323	Skin	DMSO
CRO18-LE	11.36	-16.92	Adriatic	14.4795	44.6057	Muscle	Lyophillised
CRO24-LE	13.64	-16.87	Adriatic	13.90249	44.80293	Skin	DMSO
CRO3-LE	11.67	-15.99	Adriatic	14.0436	44.8203	Lung	Lyophillised
CRO35-LE	14.55	-15.06	Adriatic	14.2443	44.6355	Skin	DMSO
CRO45-LE	15.48	-15.25	Adriatic	15.2501	44.1002	Skin	Ethanol
CRO57-LE	11.12	-16.88	Adriatic	14.8512	44.3544	Skin	DMSO
CRO74-LE	10.94	-17.22	Adriatic	14.6254	43.6801	Skin	DMSO
MAR3-LE	14.15	-16.12	Adriatic	12.8777	43.7601	Muscle	Frozen
GRE15-LE	10.51	-15.93	Aegean	23.7647	38.8019	Skin	DMSO
AZ101-LE	11.35	-17.55	Atlantic	-31.1445	39.50358	Skin	Ethanol
AZ103-LE	10.82	-17.46	Atlantic	-31.1445	39.50358	Skin	Ethanol

AZ104-LE	11.49	-17.98	Atlantic	-31.1445	39.50358	Skin	Ethanol
AZ105-LE	11.82	-17.07	Atlantic	-31.1445	39.50358	Skin	Ethanol
AZ107-LE	11.12	-19.13	Atlantic	-28.5776	38.5348	Skin	Ethanol
AZ109-LE	12.92	-17.85	Atlantic	-28.5776	38.5348	Muscle	Ethanol
AZ2-LE	13.92	-16.10	Atlantic	-28.7619	38.56228	Skin	Ethanol
AZ3-LE	13.95	-15.77	Atlantic	-28.5447	38.47889	Muscle	Ethanol
AZ41-LE	11.76	-17.08	Atlantic	-28.5776	38.5348	Muscle	Ethanol
AZ45-LE	10.00	-17.43	Atlantic	-28.5776	38.5348	Muscle	Ethanol
AZ48-LE	13.97	-15.61	Atlantic	-28.5776	38.5348	Muscle	Ethanol
AZ54-LE	10.61	-17.73	Atlantic	-28.5447	38.47889	Muscle	Ethanol
AZ7-LE	12.45	-16.00	Atlantic	-28.5776	38.5348	Muscle	Ethanol
AZ8-LE	14.59	-15.46	Atlantic	-28.5447	38.47889	Muscle	Ethanol
AZ83-LE	11.46	-18.14	Atlantic	-31.6013	37.97134	Muscle	Ethanol
AZ84-LE	11.44	-17.78	Atlantic	-31.6013	37.97134	Muscle	Ethanol
AZ90-LE	12.16	-17.57	Atlantic	-31.1445	39.50358	Muscle	Ethanol
AZ91-LE	14.00	-15.61	Atlantic	-31.1445	39.50358	Muscle	Ethanol
AZ93-LE	11.13	-17.54	Atlantic	-31.1445	39.50358	Muscle	Ethanol
AZ97-LE	12.02	-17.43	Atlantic	-31.1445	39.50358	Muscle	Ethanol
AZ99-LE	11.15	-17.50	Atlantic	-31.1445	39.50358	Muscle	Ethanol
VAL1-LE	15.05	-16.08	Balearic	-0.5048	38.17243	Skin	Frozen
VAL2-LE	14.49	-16.40	Balearic	-0.49878	38.2394	Skin	Frozen
VAL3-LE	13.89	-16.08	Balearic	-0.33322	39.41818	Skin	Frozen
VAL4-LE	12.91	-16.07	Balearic	-0.0407	38.60353	Skin	Frozen
VAL6-LE	13.88	-16.10	Balearic	-0.19996	39.66816	Skin	Frozen
VAL7-LE	15.47	-13.88	Balearic	-0.64498	38.07087	Skin	Frozen
VAL9-LE	14.37	-16.24	Balearic	-0.32241	39.47416	Skin	Frozen
VAL10-LE	14.94	-14.29	Balearic	-0.63618	38.10114	Skin	Frozen
GRE10-LE	9.96	-15.76	Ionian	23.375	38.975	Muscle	DMSO
RV3P-LE	13.21	-16.73	Estrecho	-5.55731	36.01252	Skin	DMSO
RV7M-LE	13.99	-16.60	Estrecho	-5.55731	36.01252	Skin	DMSO
RB26-LE	16.61	-14.54	Estrecho	-2.473	36.81382	Skin	DMSO
RB35-LE	15.05	-14.41	Estrecho	-5.52551	36.03065	Skin	DMSO
25-G-LE	15.65	-14.57	Gulf of Cadiz	-6.52499	36.9119	Skin	DMSO
31-G-LE	15.83	-15.11	Gulf of Cadiz	-6.52499	36.9119	Skin	DMSO
34G-LE	16.00	-14.04	Gulf of Cadiz	-6.52499	36.9119	Skin	DMSO
37G-LE	16.40	-14.18	Gulf of Cadiz	-6.52499	36.9119	Skin	DMSO
42G-LE	15.61	-14.20	Gulf of Cadiz	-6.52499	36.9119	Skin	DMSO
43G-LE	15.37	-14.28	Gulf of Cadiz	-6.52499	36.9119	Skin	DMSO
44G-LE	15.49	-14.58	Gulf of Cadiz	-6.52499	36.9119	Skin	DMSO
45G-LE	14.95	-14.59	Gulf of Cadiz	-6.52499	36.9119	Skin	DMSO
47-G-LE	16.08	-14.59	Gulf of Cadiz	-6.52499	36.9119	Skin	DMSO
48G-LE	16.49	-12.89	Gulf of Cadiz	-6.52499	36.9119	Skin	DMSO

GRE1-LE	13.17	-16.91	Ionian	20.6054	38.9324	Muscle	DMSO
PG098-LE	15.78	-15.86	Ionian	20.6054	38.9324	Skin	DMSO
SIC01-LE	11.11	-18.05	Ionian	12.64775	37.56785	Skin	DMSO
SIC02-LE	9.78	-17.00	Ionian	12.64775	37.56785	Skin	DMSO
SIC03-LE	9.45	-17.56	Ionian	12.64775	37.56785	Skin	DMSO
SIC05-LE	10.07	-16.59	Ionian	12.64775	37.56785	Skin	DMSO
SIC06-LE	10.08	-16.85	Ionian	12.64775	37.56785	Skin	DMSO
SIC09-LE	11.51	-16.39	Ionian	12.64775	37.56785	Skin	DMSO
SIC12-LE	10.24	-16.94	Ionian	12.64775	37.56785	Skin	DMSO
SIC13-LE	10.59	-16.90	Ionian	12.64775	37.56785	Skin	DMSO
TUS13-LE	14.04	-15.12	Thyrrenian	10.9089	42.6983	Muscle	Lyophillised
TUS14-LE	12.32	-16.68	Thyrrenian	10.2299	42.8391	Muscle	Lyophillised
TUS16-LE	13.10	-16.25	Thyrrenian	10.2133	43.5439	Kidney	Lyophillised
TUS17-LE	13.32	-16.22	Thyrrenian	10.2133	43.5439	Liver	Lyophillised
TUS20-LE	12.73	-16.25	Thyrrenian	10.21942	43.54789	Muscle	Lyophillised
TUS27-LE	13.68	-15.72	Thyrrenian	10.2133	43.5439	Heart	Lyophillised
TUS29-LE	12.39	-17.21	Thyrrenian	10.2151	43.8981	Liver	Lyophillised
TUS30-LE	13.24	-15.85	Thyrrenian	10.9089	42.6983	Liver	Lyophillised
TUS31-LE	13.14	-16.62	Thyrrenian	10.2133	43.5439	Liver	Lyophillised
TUS5-LE	10.68	-17.49	Thyrrenian	10.2051	42.8186	Muscle	DMSO

MOLECULAR ECOLOGY

Table S4: Pairwise F_{ST} values for the RDA comparison, outlier loci below the diagonal and neutral loci above the diagonal. Environmental variable means listed below.

	Azores	Cadiz	Alboran	Valencia	Catalonia	Tuscany	Sicily	Venice	EastAdriatic	Greece
n=	29	25	12	13	6	14	10	10	6	7
Azores	0	0.0238	0.0238	0.0141	0.0379	0.037	0.0212	0.0388	0.0386	0.0339
Cadiz	0.1036	0	0.009	0.0079	0.0094	0.0223	0.0193	0.0223	0.0223	0.0203
Alboran	0.1726	0.0508	0	0.0067	0.0066	0.0249	0.0156	0.0221	0.0197	0.0186
Valencia	0.1366	0.0552	0.0172	0	0.0138	0.0213	0.01	0.0213	0.022	0.0162
Catalonia	0.1208	0.0562	0.0054	-0.006	0	0.0242	0.0258	0.0223	0.0214	0.0265
Tuscany	0.2232	0.1459	0.0882	0.0404	0.0803	0	0.0344	0.0162	0.0113	0.0366
Sicily	0.1318	0.064	0.0603	0.0332	0.0248	0.0988	0	0.0351	0.0312	0.0242
Venice	0.2846	0.2162	0.1308	0.1204	0.1271	0.0718	0.2037	0	0.0015	0.0236
EastAdriatic	0.3106	0.2221	0.1553	0.1379	0.1565	0.0624	0.2072	0.0227	0	0.0284
Greece	0.1922	0.134	0.0782	0.0482	0.0341	0.0391	0.0883	0.0548	0.0715	0

	Azores	Cadiz	Alboran	Valencia	Catalonia	Tuscany	Sicily	Venice	EastAdriatic	Greece
SST	23.1	21.8	25	26	23.8	24.6	26.7	24	22	26.4
Salinity	36.2	36.3	36.5	37	38.1	38.2	37.6	35.5	38.5	38.8
Chl-a	0.1	1.13	0.35	0.3	0.50	0.22	0.19	1.75	0.25	0.15
$\delta^{13}C$	-17.6	-14.8	-15.1	-14.7	16.9	-17.1	-18	-16.1	-15.4	-16.3
$\delta^{15}N$	12.5	15.3	15.5	14.8	13.3	12.7	11.4	14.1	13.7	13.5

MOLECULAR ECOLOGY

Table S5: Sample metadata. Use: 1 = Genetics only, 2 = Stable isotopes only & 3 = Both

Sample	Long.	Lat.	Location	Country	Sea	Source	Sex	Date	Tissue	Storage	Sampling	Use
AZ101	-31.1445	39.50358	Flores, Azores	Portugal	Atlantic	Mónica Silva	M	11/07/2006	Skin	Ethanol	Biopsy	3
AZ103	-31.1445	39.50358	Flores, Azores	Portugal	Atlantic	Mónica Silva	M	13/07/2006	Skin	Ethanol	Biopsy	3
AZ104	-31.1445	39.50358	Flores, Azores	Portugal	Atlantic	Mónica Silva	M	13/07/2006	Skin	Ethanol	Biopsy	3
AZ105	-31.1445	39.50358	Flores, Azores	Portugal	Atlantic	Mónica Silva	M	13/07/2006	Skin	Ethanol	Biopsy	3
AZ108	-31.6013	37.97134	Princess Alice Bank	Portugal	Atlantic	Mónica Silva	M	18/08/2007	Skin	Ethanol	Biopsy	1
AZ109	-28.7619	38.56228	Faial, Azores	Portugal	Atlantic	Mónica Silva	M	23/02/2014	Muscle	Ethanol	Stranding	3
AZ2	-28.7619	38.56228	Faial, Azores	Portugal	Atlantic	Mónica Silva	M	26/04/2002	Skin	Ethanol	Biopsy	3
AZ3	-28.5447	38.47889	Pico, Azores	Portugal	Atlantic	Mónica Silva	M	03/05/2002	Muscle	Ethanol	Biopsy	3
AZ40	-28.5982	38.57719	Faial, Azores	Portugal	Atlantic	Mónica Silva	M	13/06/2003	Muscle	Ethanol	Biopsy	1
AZ41	-28.5982	38.57719	Faial, Azores	Portugal	Atlantic	Mónica Silva	M	13/06/2003	Muscle	Ethanol	Biopsy	3
AZ44	-28.5982	38.57719	Faial, Azores	Portugal	Atlantic	Mónica Silva	M	04/07/2003	Muscle	Ethanol	Biopsy	1
AZ45	-28.5982	38.57719	Faial, Azores	Portugal	Atlantic	Mónica Silva	M	04/07/2003	Muscle	Ethanol	Biopsy	3
AZ46	-28.5745	38.53961	Faial-Pico Channel	Portugal	Atlantic	Mónica Silva	M	22/07/2003	Muscle	Ethanol	Biopsy	1
AZ48	-28.5447	38.47889	Pico, Azores	Portugal	Atlantic	Mónica Silva	M	01/08/2003	Muscle	Ethanol	Biopsy	3
AZ54	-28.5447	38.47889	Pico, Azores	Portugal	Atlantic	Mónica Silva	M	15/08/2003	Muscle	Ethanol	Biopsy	3
AZ6	-28.5745	38.53961	Faial-Pico Channel	Portugal	Atlantic	Mónica Silva	F	04/05/2002	Muscle	Ethanol	Biopsy	1
AZ67	-28.5447	38.47889	Pico, Azores	Portugal	Atlantic	Mónica Silva	M	16/10/2003	Muscle	Ethanol	Biopsy	1
AZ8	-28.5447	38.47889	Pico, Azores	Portugal	Atlantic	Mónica Silva	M	01/07/2002	Muscle	Ethanol	Biopsy	3
AZ83	-31.6013	37.97134	Princess Alice Bank	Portugal	Atlantic	Mónica Silva	M	20/09/2005	Muscle	Ethanol	Biopsy	3
AZ84	-31.6013	37.97134	Princess Alice Bank	Portugal	Atlantic	Mónica Silva	M	20/09/2005	Muscle	Ethanol	Biopsy	3
AZ85	-31.6013	37.97134	Princess Alice Bank	Portugal	Atlantic	Mónica Silva	M	20/09/2005	Muscle	Ethanol	Biopsy	1
AZ86	-31.6013	37.97134	Princess Alice Bank	Portugal	Atlantic	Mónica Silva	M	20/09/2005	Muscle	Ethanol	Biopsy	1
AZ87	-31.6013	37.97134	Princess Alice Bank	Portugal	Atlantic	Mónica Silva	M	20/09/2005	Muscle	Ethanol	Biopsy	1
AZ88	-31.1445	39.50358	Flores, Azores	Portugal	Atlantic	Mónica Silva	M	06/07/2006	Muscle	Ethanol	Biopsy	1

AZ89	-31.1445	39.50358	Flores, Azores	Portugal	Atlantic	Mónica Silva	M	06/07/2006	Muscle	Ethanol	Biopsy	1
AZ92	-31.1445	39.50358	Flores, Azores	Portugal	Atlantic	Mónica Silva	M	06/07/2006	Muscle	Ethanol	Biopsy	1
AZ95	-31.1445	39.50358	Flores, Azores	Portugal	Atlantic	Mónica Silva	M	11/07/2006	Muscle	Ethanol	Biopsy	1
AZ97	-31.1445	39.50358	Flores, Azores	Portugal	Atlantic	Mónica Silva	M	11/07/2006	Muscle	Ethanol	Biopsy	3
AZ99	-31.1445	39.50358	Flores, Azores	Portugal	Atlantic	Mónica Silva	M	11/07/2006	Muscle	Ethanol	Biopsy	3
T19	-0.03074	38.59625	Altea	Spain	Balearic Sea	Alex Aguilar	M	22/04/1994	Skin	DMSO	Biopsy	1
19G	-6.85593	37.10835	Mazagon	Spain	Gulf of Cádiz	Elsa	Unk.	2005	Unk.	Unk.	Unk.	1
20GP	-6.85593	37.10835	Mazagon	Spain	Gulf of Cádiz	Elsa	Unk.	2005	Unk.	Unk.	Unk.	1
21GE	-6.85593	37.10835	Mazagon	Spain	Gulf of Cádiz	Elsa	Unk.	2005	Unk.	Unk.	Unk.	1
25GP	-6.41118	36.52347	Cádiz	Spain	Gulf of Cádiz	Elsa	Unk.	2005	Unk.	Unk.	Unk.	3
30GE	-6.41118	36.52347	Cádiz	Spain	Gulf of Cádiz	Elsa	Unk.	2005	Unk.	Unk.	Unk.	1
34GP	-6.41118	36.52347	Cádiz	Spain	Gulf of Cádiz	Elsa	Unk.	2005	Unk.	Unk.	Unk.	3
36GPC	-6.41118	36.52347	Cádiz	Spain	Gulf of Cádiz	Elsa	Unk.	2005	Unk.	Unk.	Unk.	1
37GPC	-6.41118	36.52347	Cádiz	Spain	Gulf of Cádiz	Elsa	Unk.	2005	Unk.	Unk.	Unk.	3
42G	-6.41118	36.52347	Cádiz	Spain	Gulf of Cádiz	Elsa	Unk.	2005	Unk.	Unk.	Unk.	3
43GE	-6.41118	36.52347	Cádiz	Spain	Gulf of Cádiz	Elsa	Unk.	2005	Unk.	Unk.	Unk.	3
44G	-6.41118	36.52347	Cádiz	Spain	Gulf of Cádiz	Elsa	Unk.	2005	Unk.	Unk.	Unk.	3
45GE	-6.41118	36.52347	Cádiz	Spain	Gulf of Cádiz	Elsa	Unk.	2005	Unk.	Unk.	Unk.	3
46G	-6.41118	36.52347	Cádiz	Spain	Gulf of Cádiz	Elsa	Unk.	2005	Unk.	Unk.	Unk.	1
47GE	-6.41118	36.52347	Cádiz	Spain	Gulf of Cádiz	Elsa	Unk.	2005	Unk.	Unk.	Unk.	3
RB12	-5.55731	36.01252	Estrecho	Spain	Gibralter Strait	Elsa	Unk.	2004	Unk.	Unk.	Unk.	1
RB14	-5.55731	36.01252	Estrecho	Spain	Gibralter Strait	Elsa	Unk.	2004	Unk.	Unk.	Unk.	1
RB29	-5.55731	36.01252	Estrecho	Spain	Gibralter Strait	Elsa	Unk.	2004	Unk.	Unk.	Unk.	1
RB30	-5.55731	36.01252	Estrecho	Spain	Gibralter Strait	Elsa	Unk.	2005	Unk.	Unk.	Unk.	1
RB32	-5.55731	36.01252	Estrecho	Spain	Gibralter Strait	Elsa	Unk.	2005	Unk.	Unk.	Unk.	1
RB33	-5.55731	36.01252	Estrecho	Spain	Gibralter Strait	Elsa	Unk.	2005	Unk.	Unk.	Unk.	1
RB34	-5.55731	36.01252	Estrecho	Spain	Gibralter Strait	Elsa	Unk.	2005	Unk.	Unk.	Unk.	1
RB35	-5.55731	36.01252	Estrecho	Spain	Gibralter Strait	Elsa	Unk.	2005	Unk.	Unk.	Unk.	3

RB37	-5.55731	36.01252	Estrecho	Spain	Gibralter Strait	Elsa	Unk.	2005	Unk.	Unk.	Unk.	1
RV3pE	-5.55731	36.01252	Estrecho	Spain	Gibralter Strait	CREMA	Unk.	2000	Unk.	Unk.	Unk.	3
RV7m	-5.55731	36.01252	Estrecho	Spain	Gibralter Strait	CREMA	Unk.	2000	Unk.	Unk.	Unk.	3
RB19	-2.44384	36.73353	Almería	Spain	Alborán Sea	Toftevaag	Unk.	2004	Unk.	Unk.	Unk.	1
RB2	-2.44384	36.73353	Almería	Spain	Alborán Sea	Toftevaag	Unk.	2004	Unk.	Unk.	Unk.	1
RB20	-2.44384	36.73353	Almería	Spain	Alborán Sea	Toftevaag	Unk.	2004	Unk.	Unk.	Unk.	1
RB21	-2.44384	36.73353	Almería	Spain	Alborán Sea	Toftevaag	Unk.	2004	Unk.	Unk.	Unk.	1
RB22	-2.44384	36.73353	Almería	Spain	Alborán Sea	Toftevaag	Unk.	2004	Unk.	Unk.	Unk.	1
RB23	-2.44384	36.73353	Almería	Spain	Alborán Sea	Toftevaag	Unk.	2004	Unk.	Unk.	Unk.	1
RB24E	-2.44384	36.73353	Almería	Spain	Alborán Sea	Toftevaag	Unk.	2004	Unk.	Unk.	Unk.	1
RB25	-2.44384	36.73353	Almería	Spain	Alborán Sea	Toftevaag	Unk.	2004	Unk.	Unk.	Unk.	1
RB26	-2.44384	36.73353	Almería	Spain	Alborán Sea	Toftevaag	Unk.	2004	Unk.	Unk.	Unk.	3
RB39	-3.61051	36.67973	Granada	Spain	Alborán Sea	Toftevaag	Unk.	2004	Unk.	Unk.	Unk.	1
RB4	-2.44384	36.73353	Almería	Spain	Alborán Sea	Toftevaag	Unk.	2004	Unk.	Unk.	Unk.	1
RB45	-3.03579	35.93925	Alborán	Spain	Alborán Sea	Toftevaag	Unk.	2005	Unk.	Unk.	Unk.	1
T17	-0.63618	38.10114	Guardamar	Spain	Alborán Sea	Alex Aguilar	F	15/01/1997	Muscle	Unk.	Unk.	1
T20	0.419275	40.35708	Peñíscola	Spain	Balearic Sea	Alex Aguilar	M	25/03/2000	Skin	DMSO	Unk.	1
T28	2.806675	41.66221	Blanes	Spain	Balearic Sea	Alex Aguilar	M	16/12/1993	Skin	DMSO	Unk.	1
T29	2.806675	41.66221	Blanes	Spain	Balearic Sea	Alex Aguilar	F	04/05/1993	Skin	DMSO	Unk.	1
T3	0.07384	40.00882	Castellón	Spain	Balearic Sea	Alex Aguilar	F	12/06/1992	Skin	DMSO	Unk.	1
T43	1.298667	39.0728	Balleares	Spain	Balearic Sea	Alex Aguilar	F	01/01/2001	Unk.	Unk.	Biopsy	1
T7	-0.37432	38.423	Campello	Spain	Alborán Sea	Alex Aguilar	M	06/05/1993	Skin	DMSO	Unk.	1
T8	2.132179	41.28517	Mercabarna	Spain	Balearic Sea	Alex Aguilar	F	16/12/1988	Skin	DMSO	Unk.	1
T9	3.154816	42.19014	Empúries	Spain	Balearic Sea	Alex Aguilar	F	09/04/1989	Skin	DMSO	Unk.	1
VAL1	-0.5048	38.17243	Santa Pola	Spain	Balearic Sea	Toni Raga	M	04/03/2009	Skin	Frozen	Stranding	3
VAL10	-0.63618	38.10114	Guardamar	Spain	Balearic Sea	Toni Raga	F	12/06/2014	Skin	Frozen	Stranding	3
VAL2	-0.49878	38.2394	Elche	Spain	Balearic Sea	Toni Raga	M	23/03/2011	Skin	Frozen	Stranding	3
VAL3	-0.33322	39.41818	Pinedo	Spain	Balearic Sea	Toni Raga	F	14/07/2010	Skin	Frozen	Stranding	3

MOLECULAR ECOLOGY

VAL4	-0.0407	38.60353	Altea	Spain	Balearic Sea	Toni Raga	M	11/07/2008	Skin	Frozen	Stranding	3
VAL5	-0.70174	37.95775	Torrevieja	Spain	Balearic Sea	Toni Raga	M	25/06/2010	Skin	Frozen	Stranding	1
VAL6	-0.19996	39.66816	Sagunto	Spain	Balearic Sea	Toni Raga	F	19/03/2011	Skin	Frozen	Stranding	3
VAL7	-0.64498	38.07087	Orihuela	Spain	Balearic Sea	Toni Raga	F	18/08/2017	Skin	Frozen	Stranding	3
CL17	11.18337	42.46276	Orbetello	Italy	Thyrrenian	Marsili Letizia	F	17/11/1996	Skin	Frozen	Stranding	1
CL551	10.50248	43.28946	Tuscany	Italy	Thyrrenian	Marsili Letizia	M	26/06/1990	Skin	Frozen	Stranding	1
CL59	10.29758	43.53617	Livorno	Italy	Thyrrenian	Marsili Letizia	Unk.	24/05/1990	Heart	Frozen	Stranding	1
GB183	12.56938	37.65721	Mazara Del Vello	Italy	Mediterranean	Giusi Buscaino	F	10/05/2000	Skin	DMSO	Stranding	1
SIC01	12.64775	37.56785	Torretta Granitola	Italy	Mediterranean	Daniel Moore	Unk.	06/09/2017	Skin	DMSO	Biopsy	3
SIC02	12.64775	37.56785	Torretta Granitola	Italy	Mediterranean	Daniel Moore	Unk.	06/09/2017	Skin	DMSO	Biopsy	3
SIC03	12.64775	37.56785	Torretta Granitola	Italy	Mediterranean	Daniel Moore	Unk.	07/09/2017	Skin	DMSO	Biopsy	3
SIC05	12.64775	37.56785	Torretta Granitola	Italy	Mediterranean	Daniel Moore	Unk.	07/09/2017	Skin	DMSO	Biopsy	3
SIC06	12.64775	37.56785	Torretta Granitola	Italy	Mediterranean	Daniel Moore	Unk.	07/09/2017	Skin	DMSO	Biopsy	3
SIC09	12.64775	37.56785	Torretta Granitola	Italy	Mediterranean	Daniel Moore	Unk.	10/09/2017	Skin	DMSO	Biopsy	3
SIC12	12.64775	37.56785	Torretta Granitola	Italy	Mediterranean	Daniel Moore	Unk.	12/09/2017	Skin	DMSO	Biopsy	3
SIC13	12.64775	37.56785	Torretta Granitola	Italy	Mediterranean	Daniel Moore	Unk.	18/09/2017	Skin	DMSO	Biopsy	3
TUS28	10.50248	43.28946	Tuscany	Italy	Thyrrenian	Ada Natoli	M	27/06/1994	Skin	Frozen	Stranding	1
TtLAZ1	12.22375	41.76242	Rome	Italy	Thyrrenian	MMTB	M	30/06/2011	Muscle	DMSO	Stranding	1
TtSIC3	12.5794	37.6401	Mazara Del Vello	Italy	Mediterranean	Giusi Buscaino	F	10/06/2004	Skin	DMSO	Stranding	1
TtSIC4	12.58529	35.52302	Lampedusa	Italy	Mediterranean	Uni. of Siena	M	01/07/2006	Skin	DMSO	Stranding	1
TtTUS1	10.1851	43.8582	Viareggio	Italy	Thyrrenian	Uni. of Siena	Unk.	13/03/2008	Skin	DMSO	Stranding	1
TtTUS10	10.2133	43.5439	Livorno	Italy	Thyrrenian	MMTB	M	17/12/2009	Muscle	DMSO	Stranding	1
TtTUS14	10.2299	42.8391	Elba	Italy	Thyrrenian	Uni. of Siena	M	08/06/1999	Muscle	Lyophillised	Stranding	3
TtTUS2	10.1851	43.8582	Viareggio	Italy	Thyrrenian	Uni. of Siena	Unk.	14/03/2008	Skin	DMSO	Stranding	1
TtTUS20	10.21942	43.54789	Meloria	Italy	Thyrrenian	Uni. of Siena	F	24/05/1990	Muscle	Lyophillised	Stranding	3
TtTUS3	10.2133	43.5439	Livorno	Italy	Thyrrenian	Uni. of Siena	Unk.	17/12/2009	Skin	DMSO	Unk.	1
TtTUS38	11.1414	42.4554	Orbetello	Italy	Thyrrenian	Ada Natoli	F	18/11/2000	Unk.	Frozen	Stranding	1
TtTUS4	11.09336	42.43067	Monte Argentario	Italy	Thyrrenian	Banca Tessuti	M	17/05/2007	Muscle	DMSO	Unk.	1

TtTUS7	10.10941	43.99943	Antignano	Italy	Thyrrenian	Banca Tessuti	F	13/03/2008	Muscle	DMSO	Unk.	1
TtTUS8	10.10941	43.99943	Antignano	Italy	Thyrrenian	Banca Tessuti	F	14/03/2008	Muscle	DMSO	Unk.	1
TtTUS9	10.48984	43.29798	Marina di Cecina	Italy	Thyrrenian	Banca Tessuti	M	07/10/2008	Muscle	DMSO	Unk.	1
2_97	20.8702	38.6201	Kalamos	Greece	Aegean	Stefania Gaspari	M	29/08/1996	Teeth	Unk.	Stranding	1
2_99	20.8702	38.6201	Kalamos	Greece	Aegean	Ada Natoli/TRI	F	21/06/1999	Skin	DMSO	Biopsy	1
3_97	20.8702	38.6201	Kalamos	Greece	Aegean	Ada Natoli/TRI	M	29/08/1997	Teeth	Unk.	Stranding	1
6_97	20.8702	38.6201	Kalamos	Greece	Aegean	Ada Natoli/TRI	F	11/10/2001	Skin	DMSO	Biopsy	1
CL529	12.30326	45.21086	Chioggia	Italy	Adriatic	Marsili Letizia	Unk.	16/08/1992	Skin	Frozen	Unk.	1
CL540	12.94528	43.89593	Pesaro	Italy	Adriatic	Marsili Letizia	M	16/08/1992	Skin	Frozen	Stranding	1
CL541	12.3676	44.24975	Forli	Italy	Adriatic	Marsili Letizia	F	20/08/1992	Skin	Frozen	Stranding	3
CL542	12.39737	44.20935	Cesanatico	Italy	Adriatic	Marsili Letizia	F	11/04/1992	Skin	Frozen	Stranding	1
EPLIDO	12.37673	45.40981	Lido Venezia	Italy	Adriatic	Tethys	F	2000	Skin	DMSO	Unk.	1
FILIPPO	15.90232	41.60957	Manfredonia	Italy	Adriatic	Tethys	M	30/10/1998	Skin	DMSO	Unk.	1
G20	14.64953	44.69651	Unk.	Croatia	Adriatic	Tethys	F	12/10/2001	Muscle	Lyophillised	Stranding	1
INCOGNI.	14.64953	44.69651	Unk.	Croatia	Adriatic	Ada Natoli/TRI	Unk.	Unk.	Skin	DMSO	Stranding	1
T13	0.590146	40.60643	San Carles	Spain	Balearic	Alex Aguilar	F	1994	Skin	DMSO	Unk.	1
T16	-0.57232	38.18723	Santa Pola, Alicante	Spain	Alborán	Alex Aguilar	M	1992	Skin	DMSO	Unk.	1
T21	-0.2287	38.50012	Villajoyosa	Spain	Alborán	Alex Aguilar	F	1992	Skin	DMSO	Unk.	1
TRI006	14.39224	44.69649	Osor, Island of Cres	Croatia	Adriatic	Tethys	M	20/10/1994	Skin	DMSO	Stranding	1
TRI011	14.24478	44.6357	Unije Island	Croatia	Adriatic	Tethys	F	31/07/1997	Skin	DMSO	Stranding	1
TRI014	14.64953	44.69651	Unk.	Croatia	Adriatic	Tethys	F	13/07/1999	Skin	DMSO	Stranding	1
TUR1	20.8702	38.6201	Kalamos	Greece	Aegean	Tethys	M	22/08/1993	Skin	DMSO	Stranding	1
TURC1	20.8702	38.6201	Kalamos	Greece	Aegean	Tethys	F	09/10/1993	Skin	DMSO	Biopsy	1
TtGRE1	20.6054	38.9324	Mytikas	Greece	Ionian	Banca Tessuti	F	03/09/2007	Muscle	DMSO	Stranding	3
TtGRE14	20.87535	37.72433	Zakynthos	Greece	Ionian	Alexadrox Fazis	M	22/07/2009	Skin	DMSO	Unk.	1
TtGRE18	20.8702	38.6201	Kalamos	Greece	Aegean	Ada Natoli	M	22/08/1993	Skin	DMSO	Unk.	1
TtGRE20	20.8702	38.6201	Kalamos	Greece	Aegean	Ada Natoli	M	22/08/1994	Skin	DMSO	Stranding	1
TtMAR1	12.7901	43.9341	Pesaro	Italy	Adriatic	Marsili Letizia	M	16/08/1992	Skin	Frozen	Unk.	1

TtPUG4	16.1861	41.5865	Manfredonia	Italy	Adriatic	Tethys	M	30/10/1998	Skin	Frozen	Unk.	1
TtROM18	12.3201	44.3444	Cervia	Italy	Adriatic	MMTB	M	17/05/2011	Muscle	DMSO	Unk.	1
TtROM19	12.5088	44.1226	Rimini	Italy	Adriatic	MMTB	M	06/07/2011	Muscle	DMSO	Unk.	1
TtROM7	12.3233	44.2922	Forli	Italy	Adriatic	Marsili Letizia	F	20/08/1992	Skin	Frozen	Unk.	1
TtROM9	12.3273	44.2921	Cesanatico	Italy	Adriatic	Ada Natoli	F	12/04/1996	Skin	Frozen	Stranding	1
TtSLO1	13.5542	45.5398	Unk.	Slovenia	Adriatic	Tilen Genov	M	03/09/2011	Skin	Ethanol	Biopsy	1
TtSLO3	13.5542	45.5398	Unk.	Slovenia	Adriatic	Tilen Genov	M	07/09/2011	Skin	Ethanol	Biopsy	1
TtSLO5	13.5542	45.5398	Unk.	Slovenia	Adriatic	Tilen Genov	M	07/09/2011	Skin	Ethanol	Biopsy	1
TtSLO7	13.5542	45.5398	Unk.	Slovenia	Adriatic	Tilen Genov	F	07/09/2011	Skin	Ethanol	Biopsy	1
TtVEN1	12.48472	45.35694	Chioggia	Italy	Adriatic	Marsili Letizia	M	16/08/1992	Skin	Frozen	Unk.	1
TtVEN13	12.35111	45.10274	Rosolina	Italy	Adriatic	MMTB	F	09/09/2010	Muscle	DMSO	Unk.	1
Tur2	20.91822	38.60966	Kalamos	Greece	Ionian Sea	Tethys	M	22/08/1994	Skin	DMSO	Stranding	1
BS10	35.24917	44.91632	Kara Dag Reserve	Russia	Black Sea	Alexei Birkun	F	24/03/2002	Skin	DMSO	Unk.	1
BS11	35.24917	44.91632	Kara Dag Reserve	Russia	Black Sea	Alexei Birkun	M	24/03/2002	Skin	DMSO	Unk.	1
BS2	34.35758	44.57537	Partenit	Russia	Black sea	Alexei Birkun	M	12/05/2002	Skin	DMSO	Unk.	1
BS3	34.17402	44.48895	Yalta	Russia	Black Sea	Alexei Birkun	M	12/05/2002	Skin	DMSO	Unk.	1
BS4	34.17402	44.48895	Yalta	Russia	Black Sea	Alexei Birkun	F	12/05/2002	Skin	DMSO	Unk.	1
BS5	33.37168	45.17442	Evpatoria	Russia	Black Sea	Alexei Birkun	M	17/03/2002	Skin	DMSO	Unk.	1
BS6	33.37168	45.17442	Evpatoria	Russia	Black Sea	Alexei Birkun	M	17/03/2002	Skin	DMSO	Unk.	1
BS7	33.37168	45.17442	Evpatoria	Russia	Black Sea	Alexei Birkun	M	17/03/2002	Skin	DMSO	Unk.	1
BS8	33.37168	45.17442	Evpatoria	Russia	Black Sea	Alexei Birkun	F	17/03/2002	Skin	DMSO	Unk.	1
BS1	34.35758	44.57537	Partenit	Russia	Black sea	Alexei Birkun	M	12/05/2002	Skin	DMSO	Unk.	1
CRO101	14.4795	44.6057	Punta Kriza	Croatia	Adriatic	BWI - Drasko	F	24/08/2010	Skin	DMSO	Unk.	2
CRO24	14.0436	44.8203	Premantura	Croatia	Adriatic	BWI - Drasko	F	21/01/2000	Skin	DMSO	Unk.	2
CRO35	14.2443	44.6355	Sesnja	Croatia	Adriatic	Ada Natoli	Unk.	29/08/2001	Skin	DMSO	Stranding	2
CRO45	15.2501	44.1002	Zabodarski	Croatia	Adriatic	BWI - Drasko	Unk.	14/07/2003	Skin	Ethanol	Unk.	2
CRO57	14.8512	44.3544	Silba	Croatia	Adriatic	BWI - Drasko	Unk.	17/08/2006	Skin	DMSO	Unk.	2
CRO74	14.6254	43.6801	Silba	Croatia	Adriatic	BWI - Drasko	M	06/11/2007	Skin	DMSO	Unk.	2

MOLECULAR ECOLOGY

MAR1	12.8777	43.7601	S. Bartolo	Italy	Adriatic	Ada Natoli	M	05/07/1996	Muscle	Frozen	Stranding	2
CRO18	14.4795	44.6057	Unk.	Croatia	Adriatic	Ada Natoli	F	31/07/1997	Muscle	Lyophillised	Stranding	2
AZ107	-28.5776	38.5348	Unk.	Portugal	Atlantic	Mónica Silva	Unk.	Unk.	Skin	Ethanol	Biopsy	2
AZ7	-28.5776	38.5348	Unk.	Portugal	Atlantic	Mónica Silva	Unk.	Unk.	Muscle	Ethanol	Biopsy	2
AZ90	-28.5776	38.5348	Unk.	Portugal	Atlantic	Mónica Silva	Unk.	Unk.	Muscle	Ethanol	Biopsy	2
AZ91	-28.5776	38.5348	Unk.	Portugal	Atlantic	Mónica Silva	Unk.	Unk.	Muscle	Ethanol	Biopsy	2
AZ93	-28.5776	38.5348	Unk.	Portugal	Atlantic	Mónica Silva	Unk.	Unk.	Muscle	Ethanol	Biopsy	2
GRE15	23.7647	38.8019	L.V. Attikis	Greece	Aegean	Alexadrox Fazis	M	17/10/2009	Skin	DMSO	Unk.	2
GRE10	23.375	38.975	Iraklion	Greece	Aegean	Alexadrox Fazis	Unk.	10/07/2006	Muscle	DMSO	Unk.	2
TUS5	10.2051	42.8186	Marciana	Italy	Thyrrenian	Banca Tessuti	F	03/10/2007	Muscle	DMSO	Unk.	2
VAL9	-0.32241	39.47416	Guardamar del Segura	Spain	Balearic	Toni Raga	M	11/07/2008	Muscle	Frozen	Stranding	2
CL546	12.3063	44.4598	Ravenna	Italy	Thyrrenian	Ada Natoli	M	24/08/1996	Skin	Frozen	Stranding	2
31G	-6.52499	36.9119	Unk.	Spain	S of Gulf of Cadiz	Elsa	Unk.	2005	Unk.	Unk.	Unk.	2
48G	-6.52499	36.9119	Unk.	Spain	S of Gulf of Cadiz	Elsa	Unk.	2005	Unk.	Unk.	Unk.	2
TUS13	10.9089	42.6983	Marina di Grosseto	Italy	Thyrrenian	Ada Natoli/Siena	F	24/03/2002	Unk.	Lyophillised	Unk.	2
TUS16	10.2133	43.5439	Livorno	Italy	Thyrrenian	Ada Natoli/Siena	F	26/07/1991	Kidney	Lyophillised	Unk.	2
TUS17	10.2133	43.5439	Livorno	Italy	Thyrrenian	Ada Natoli/Siena	F	05/05/1990	Liver	Lyophillised	Unk.	2
TUS27	10.2133	43.5439	Livorno	Italy	Thyrrenian	Ada Natoli/Siena	F	25/05/1994	Heart	Lyophillised	Stranding	2
TUS29	10.2151	43.8981	Lido di Camaiore	Italy	Thyrrenian	Ada Natoli/Siena	F	11/04/1994	Liver	Lyophillised	Stranding	2
TUS30	10.9089	42.6983	Marina di Grosseto	Italy	Thyrrenian	Ada Natoli/Siena	M	19/05/1995	Liver	Lyophillised	Stranding	2
TUS31	10.2133	43.5439	Livorno	Italy	Thyrrenian	Ada Natoli/Siena	M	27/07/1995	Liver	Lyophillised	Stranding	2
TUS5	10.2051	42.8186	Marciana Marina	Italy	Thyrrenian	Ada Natoli/Siena	F	03/10/2007	Muscle	DMSO	Unk.	2

Reference:

Masson-Delmotte, V., Stenni, B., Pol, K., Braconnot, P., Cattani, O., Falourd, S., Kageyama, M., Jouzel, J., Landais, A., Minster, B. and Barnola, J.M., 2010. EPICA Dome C record of glacial and interglacial intensities. *Quaternary Science Reviews*, *29*(1-2), pp.113-128.
